# Measurement of 100 nm and 60 nm Particle Standards by Differential Mobility Analysis

**DOI:** 10.6028/jres.111.022

**Published:** 2006-08-01

**Authors:** George W. Mulholland, Michelle K. Donnelly, Charles R. Hagwood, Scott R. Kukuck, Vincent A. Hackley, David Y. H. Pui

**Affiliations:** National Institute of Standards and Technology, Gaithersburg, MD 20899; Department of Mechanical Engineering, Particle Technology Laboratory, University of Minnesota, Minneapolis, MN 55455

**Keywords:** differential mobility analysis, dynamic light scattering, electrical mobility, electrospray aerosol generation, particle size calibration standards, transfer function

## Abstract

The peak particle size and expanded uncertainties (95 % confidence interval) for two new particle calibration standards are measured as 101.8 nm ± 1.1 nm and 60.39 nm ± 0.63 nm. The particle samples are polystyrene spheres suspended in filtered, deionized water at a mass fraction of about 0.5 %. The size distribution measurements of aerosolized particles are made using a differential mobility analyzer (DMA) system calibrated using SRM^®^ 1963 (100.7 nm polystyrene spheres). An electrospray aerosol generator was used for generating the 60 nm aerosol to almost eliminate the generation of multiply charged dimers and trimers and to minimize the effect of non-volatile contaminants increasing the particle size. The testing for the homogeneity of the samples and for the presence of multimers using dynamic light scattering is described. The use of the transfer function integral in the calibration of the DMA is shown to reduce the uncertainty in the measurement of the peak particle size compared to the approach based on the peak in the concentration vs. voltage distribution. A modified aerosol/sheath inlet, recirculating sheath flow, a high ratio of sheath flow to the aerosol flow, and accurate pressure, temperature, and voltage measurements have increased the resolution and accuracy of the measurements. A significant consideration in the uncertainty analysis was the correlation between the slip correction of the calibration particle and the measured particle. Including the correlation reduced the expanded uncertainty from approximately 1.8 % of the particle size to about 1.0 %. The effect of non-volatile contaminants in the polystyrene suspensions on the peak particle size and the uncertainty in the size is determined. The full size distributions for both the 60 nm and 100 nm spheres are tabulated and selected mean sizes including the number mean diameter and the dynamic light scattering mean diameter are computed. The use of these particles for calibrating DMAs and for making deposition standards to be used with surface scanning inspection systems is discussed.

## 1. Introduction

The National Institute of Standards and Technology (NIST) issues Standard Reference Materials (SRM^®^) for a wide range of particle sizes including 100 nm (SRM^®^ 1963), 0.3 µm (SRM^®^ 1691), 1 µm (SRM^®^ 1690), 3 µm (SRM^®^ 1692), 10 µm (SRM^®^ 1960), and 30 µm (SRM^®^ 1961). These standards are monosize polystyrene spheres suspended in water at a mass fraction in the range 0.5 % to 1 %.

The focus of this paper is the measurement results and uncertainty analyses for two batches of particles with nominal sizes of 100 nm and 60 nm. The 60 nm size is needed in the calibration of scanning surface inspection systems for the minimum particle size detected. These devices are used in the semiconductor industry to measure the number of contaminant particles of size 60 nm and larger on a bare silicon wafer. The 100 nm particle discussed here is intended to replace SRM^®^ 1963, for which individual spheres in many of the previously prepared vials have formed agglomerates. In many cases a large floc was visible to the eye in the 5 mL vials. Quantitative evidence for the presence of dimers and trimers was obtained by Fitzpatrick [1] using a disk centrifuge. Five samples were analyzed, and, in four of the five, the fraction of multimers exceeded 30 %. The presence of multimers was also evident from dynamic light scattering (DLS) measurements. While the individual peaks were not resolved, the peak size as interpreted by DLS was typically 30 % to 70 % greater than the nominal monomer particle size.

When evidence of agglomeration was first quantified in SRM^®^ 1963, a decision was made to modify the Certification of Analysis to state that “the standard is not appropriate for applications where monosize, unagglomerated spheres are necessary.” The samples were still useful for calibration of electron microscopes and for generating a monosize aerosol using a differential mobility classifier (DMA), since there was still a high enough concentration of monomers present. However, there was evidence from the semiconductor industry that the use of SRM^®^ 1963 particles as a deposition standard in an agglomerated state was problematic. Because of the importance of the 100 nm size range in the semiconductor industry for the calibration of surface scanning inspection systems and because of the need for unagglomerated spheres in calibrating optical scattering instruments such as dynamic light scattering instruments, a decision was made to issue a replacement for SRM^®^ 1963.

The general approach to the measurement of particle size and measurement uncertainty is similar to that used by Mulholland *et al.* [2]. In the current study, SRM^®^ 1963 was used for calibrating the differential mobility analyzer (DMA), while in the earlier study [2] a mono-size aerosol with a number mean size of 895 nm (SRM^®^ 1690) was used to calibrate the classifier. Among the remaining SRM^®^ 1963 samples, several unagglomerated samples were found and used for the calibration.

The calibration approach and measurement method have been modified since the earlier study [2] to account for the effect of the finite width of the DMA transfer function on the measured peak particle size. This approach, which is similar to that of Ehara *et al.* [3], is used to assess the error resulting from the use of the simpler approach in [2]. The theoretical approach and the numerical methods used are presented in Sec. 2.

The physical properties used to measure the particle size including slip correction, electron charge, charging probability as a function of particle diameter, viscosity, and mean free path are presented in Sec. 3 along with the formulas used to compute the quantities for a range of conditions. The estimated uncertainties in the properties are included.

There have been several improvements in the instrumentation since the earlier study. The use of a modified aerosol/sheath inlet, a recirculating sheath flow, and a 40 to 1 ratio of sheath flow to the aerosol flow increased the resolution and accuracy of the measurements. The uncertainty in the pressure and temperature measurement have been reduced by at least a factor of ten and the uncertainty in the DMA voltage has been reduced by almost a factor of two for the 100 nm sphere measurements. In addition, a pneumatic nebulizer with a more constant output was used for the 100 nm spheres and an electrospray generator was used for the 60 nm spheres to reduce the effects of multiply charged multimers and nonvolatile impurities in the particle-water suspension. These new features together with the general measurement approach are presented in Sec. 4. The characteristics of the 100 nm spheres and 60 nm spheres are presented in Sec. 5 along with a description of the sample preparation for use with the DMA measurement system.

A number of samples were selected at random with repeat measurements made on each sample to assess the homogeneity of the samples in terms of the peak particle size. A series of measurements were then made on a single sample to determine the peak particle size based on at least three repeat measurements over each of three different days. For every sample measurement there was also a calibration measurement. The measurement approach and analysis are described in Sec. 6 and the experimental design, statistical test for sample homogeneity, and the analysis of the Type A uncertainty [4], which is computed by statistical methods, are presented in Sec. 7.

The Type B uncertainty analysis, which is usually based on scientific judgment, is presented in Sec. 8. One significant improvement in the uncertainty analysis is accounting for the correlation between the slip correction for the measured particle, either the 60 nm or 100 nm, and the slip correction of the SRM^®^ 1963 particles used in calibrating the DMA. Other important considerations in the uncertainty analysis are the drift in the number concentration during the measurement of the number concentration versus voltage, the overlap between singlet monomers and doublet trimers, and the presence of non-volatile contaminants in the polystyrene sphere suspension. The 100 nm and 60 nm sphere diameters as aerosols are corrected for the residuer layer on the SRM^®^ 1963 spheres.

The results of dynamic light scattering to assess the presence of multiplets in the sample vials are presented in Sec. 9. Dynamic light scattering will be used in the future to verify that agglomeration has not taken place within the sample. Transmision electron microscopy results for the 60 and 100 nm spheres are presented in Sec. 10.

The particle size measured in this study is the modal diameter, i.e. the peak in the number size distribution. The choice of the modal diameter for certification is motivated by the wide use of DMA’s in the deposition of monodisperse particles on wafers, operated at the voltage corresponding to the peak transmission (throughput). Results are also provided in Sec. 11 for the number mean diameter and the so called Z-averaged diameter measured by dynamic light scattering. The full size distribution for both the 60 nm and 100 nm spheres is tabulated. Also in Sec. 11, the estimated size distribution of particles deposited on a wafer resulting from operating a DMA at the peak voltage for the 60 nm spheres or 100 nm spheres is presented.

## 2. Theoretical Background

The approach used in this study is to determine the size distribution of particles by measuring their electrical mobility, *Z_p_*. The relationship between particle diameter, *D_p_*, and electrical mobility can be obtained by performing a balance between the electric force, assumed to be in the x direction, and the drag force on a singly charged particle initially at rest.

$$eE_{x}=3\pi \eta D_{p}v_{x}/C(D_{p}),$$ (1)

where *η* is the gas viscosity, *e* is the electron charge, and *C*(*D_p_*) is the Cunningham slip correction factor that corrects for non-continuum gas behavior on the drag force for small particles. The particle will initially accelerate in response to the electric field *E_x_* and approach the velocity *v_x_* for which the drag force balances the electric force. Solving for the ratio of the velocity to the electric field, the definition of *Z_p_*, one obtains an expression for *Z_p_* as a function of particle diameter:

$$Z_{p}=\frac{eC(D_{p})}{3\pi \eta D_{p}}.$$ (2)

This equation provides the basis for measuring the particle size distribution via electrical mobility measurements. The size dependence of the electrical mobility ranges from an inverse dependence on diameter for sizes large compared to the mean free path of the gas (Stokes limit) to an inverse quadratic dependence for particle sizes much smaller than the mean free path (free molecular limit).

**Fig. 1:**
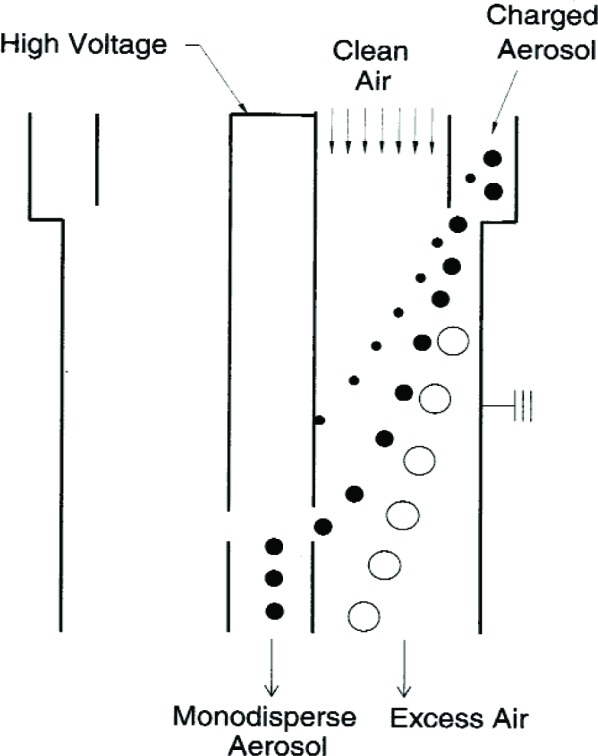
Schematic diagram of the DMA. As the polydisperse aerosol flows through a DMA, a monodisperse fraction exits through the central electrode as a result of the size dependence of the electrical mobility.

The study makes use of an annular differential mobility analyzer (DMA), illustrated in Fig. 1. The DMA consists of an inner cylindrical rod with radius *r*_1_ connected to a variable high voltage DC power supply with voltage *V*, and an outer annular tube with radius *r*_2_ connected to ground. Before entering the DMA a particle goes through a bipolar charger. Clean sheath air flows through the axial region at a flow rate *Q_c_*, while the charged aerosol enters at a flow rate *Q_a_* through an axisymmetric opening along the outer cylinder. The distance between the midpoint of the inlet and the midpoint of the exit is *L*. The positively charged particles move radially towards the center rod under the influence of the electric field. Near the bottom of the classifying region, a flow consisting of nearly-monodisperse aerosol exits through a slit in the center rod at a flow rate *Q_a_*.

### 2.1 Convolution Integral and DMA Transfer Function

Knutson and Whitby [5] derived an expression for the number of particles, *N*(*V*), exiting the DMA at voltage *V* involving an integral over the product of the DMA transfer function *Ω* and the mobility size distribution function *F*(*Z_p_*), where *F*(*Z_p_*)*dZ_p_* is equal to the number of particles with mobility between *Z_p_* and *Z_p_*+ *dZ_p_*. The result for the case where the inlet aerosol flow is equal to the outlet flow is given by:

$$N(V)=\int_{0}^{\infty }\Omega (Z_{p}\cdot V)F(Z_{p})dZ_{p}.$$ (3)

It is assumed that *N*(*V*) is the part of the DMA spectrum for singly charged particles, and it is also assumed that the size distribution is relatively narrow with a standard deviation less than 15 % of the peak diameter, so that only singly charged particles are present at the peak. The transfer function *Ω* for the DMA operating at voltage *V* is defined as the probability that a charged particle entering the DMA with electric mobility *Z_p_* will leave through the sampling slit. The transfer function *Ω* has a triangular shape with a peak value of 1 and, for a perfectly monodisperse aerosol, all the aerosol entering the DMA exits through the slit in the center electrode for the voltage corresponding to the peak in the transfer function.

Our primary interest is in obtaining the diameter size distribution, *G*(*D_p_*), where *G*(*D_p_*)*dD_p_* is equal to the number of particles per cm^3^ with diameter between *D_p_* and *D_p_*+ *dD_p_*. In one case, we will use a known diameter distribution to calibrate the DMA and, in another case, we will be using the DMA to measure the peak in the diameter distribution for two new particle size calibration standards. The relationship between *F*(*Z_p_*) and *G*(*D_p_*) is given by:

$$F(Z_{p})=G(D_{p}(Z_{p}))p(D_{p}(Z_{p}))\left|dD_{p}/dZ_{p}\right|.$$ (4)

The quantity *p*(*D_p_*) is the probability that a particle with diameter *D_p_* carries one elementary unit of charge. The absolute value of the derivative in Eq. (4) reflects the fact that *F*(*Z_p_*) and *G*(*D_p_*) are positive definite quantities but the inverse dependence of the mobility on the diameter results in a negative derivative.

Substituting the expression for *F*(*Z_p_*) into Eq. (3), we obtain:

$$N(V)=\int_{0}^{\infty }\Omega (Z_{p}\cdot V)G(D_{p}(Z_{p}))p(D_{p}(Z_{p}))\left|dD_{p}/dZ_{p})\right|dZ_{p}.$$ (5)

It is convenient when carrying out the numerical integration of Eq. (5) for a given size distribution function to express the integral in terms of the dimensionless mobility *x* defined as:

$$x=\frac{2\pi \Lambda Z_{p}V}{Q_{c}}.$$ (6)

Here, *Λ* is a geometric factor based on the inner and outer radius and the length of the classifying region of the DMA,

$$\Lambda =L/\ln(r_{2}/r_{1}).$$ (7)

The transfer function *Ω* can be conveniently expressed in terms of both *x* and the ratio of the aerosol flow to the sheath flow, *δ* = *Q_a_*/*Q_c_*,

$$\begin{array}{ll} \Omega (x)=0 & x<1-\delta \\ \Omega (x)=1-\frac{\left(1-x\right)}{\delta } & 1-\delta \leq x<1\\ \Omega (x)=1+\frac{\left(1-x\right)}{\delta } & 1\leq x\leq 1+\delta \\ \Omega (x)=0 & x>1+\delta . \end{array}$$ (8)

The transfer function is triangular and increases from zero to 1 and then decreases back to zero over a range of *x* equal to 2*δ*. The standard deviation of the transfer function divided by the average value of *x*, $\sigma_{r}^{T}(x)$, is equal to $\delta /\sqrt{6}$. This provides a convenient dimensionless measure of the DMA resolution in terms of mobility. One can see from the definition of *δ* that the measurement resolution increases as the value of the ratio of the aerosol flow to sheath flow is decreased. In the measurements described below, the value of *δ* was 0.025, corresponding to a sheath flow of 20 L/min and an aerosol flow of 0.5 L/min. For this value of *δ*, $\sigma_{r}^{T}(x)=0.0102$.

The standard deviation of the transfer function expressed in terms of diameter is a more convenient value for comparison with the standard deviation of the size distribution. From Eqs. (2) and (6), one can derive the following relationship:

$$\frac{dD_{p}}{D_{p}}=\frac{dx}{x}{\left(\frac{C^{\prime }(D_{p})D_{p}}{C(D_{p})}-1\right)}^{-1}.$$ (9)

Replacing the reduced differentials with the corresponding standard relative deviations, one obtains

$$\sigma_{r}^{T}(D_{p})=\sigma_{r}^{T}(x){\left(1-\frac{C^{\prime }(D_{p})D_{p}}{C(D_{p})}\right)}^{-1}.$$ (10)

Assuming nominal temperature and pressure of the measurement condition to be 23 °C and 101.33 kPa, respectively, the computed value of $\sigma_{r}^{T}(D_{p})$ is 0.0058 for the 101.6 nm spheres and 0.0055 for the 60.5 nm spheres.

With the transfer function expressed in terms of *x*, it is also convenient to express the integral in Eq. (5) as an integral over *x*. From Eq. (9), one can also derive the following relationship:

$$\left|dD_{p}/dZ_{p}\right|dZ_{p}=\frac{1}{x}{\left|\frac{C^{\prime }(D_{p})}{C(D_{p})}-\frac{1}{D_{p}}\right|}^{-1}dx.$$ (11)

Using Eq. (8) and Eq. (11), the integral can then be expressed in terms of *x* with the result:

$$\begin{array}{l} N(V)=\int_{1-\delta }^{1}\left(1-\frac{\left(1-x\right)}{\delta }\right)G(D_{p}(x))p(D_{p}(x))\\ \;\frac{1}{x}{\left|\frac{C^{\prime }(D_{p})}{C(D_{p})}-\frac{1}{D_{p}}\right|}^{-1}dx+\int_{1}^{1+\delta }\left(1+\frac{\left(1-x\right)}{\delta }\right)\\ \;G(D_{p}(x))p(D_{p}(x))\frac{1}{x}{\left|\frac{C^{\prime }(D_{p})}{C(D_{p})}-\frac{1}{D_{p}}\right|}^{-1}dx. \end{array}$$ (12)

A computer program was written to carry out the integration. The integrations were carried out both for calibrating the DMA and for estimating the accuracy of an approximate method given below for determining the peak particle size for an unknown size distribution. The expression used for the slip correction is given in Sec. 3.

### 2.2 Calibration of DMA and Determination of Peak Particle Size

The DMA is calibrated using the accurately sized SRM^®^ 1963 with *G*(*D_p_*) assumed Gaussian and having a mean size of 100.7 nm and an estimated standard deviation equal to 2.0 nm. The number concentration is measured versus voltage and the peak voltage is determined. This value is compared with the prediction of Eq. (12) where the integration limits are set by the flow ratio of *δ* = 0.025. If the sheath flow, *Q_c_*, used in the prediction differs from the actual flow, then the measured peak voltage will not agree with the predicted peak voltage. It is seen from Eq. (6), however, that the flow rate *Q_c_* can be adjusted by the ratio of the measured voltage to the predicted voltage so that the mobility *Z_p_* is the same for both the measurement and the predicted value. This adjustment, therefore, calibrates the parameters used in the calculations to actual operational conditions.

It is also possible that the geometric factor is in error. In this case, the above correction would be a combined correction factor for both the flow and the geometric factor. The basis for using this approach rather than using the directly measured flow and geometric parameter is that the combined uncertainty is lower using an accurately known calibration standard versus using the measured values together with uncertainty in the flow and in the geometric factor.

#### 2.2.1 Approximation 1

In general, the determination of the size distribution requires the inversion of Eq. (12). For the case in which the size distribution is broad and changing slowly with diameter, an approximate expression can be obtained for *G*(*D_p_*). In this case, the transfer function varies much more rapidly with *x* than do the other functions appearing in the integrand of Eq. (12). The other functions are, therefore, evaluated at the value of *D_p_* corresponding to the peak in the transfer function, *x* = 1, for the given voltage. This leads to the following result:

$$\begin{array}{c} N(V)=p(D_{p}(x=1))G(D_{p}(x=1)){\left({\left|\frac{C^{\prime }(D_{p})}{C(D_{p})}-\frac{1}{D_{p}}\right|}_{x=1}\right)}^{-1}\\ \times \left[\int_{1-\delta }^{1}\left(1-\frac{\left(1-x\right)}{\delta }\right)dx+\int_{1}^{1+\delta }\left(1-\frac{\left(1-x\right)}{\delta }\right)dx\right]. \end{array}$$ (13)

The integral of the transfer function is *δ*, simply the area of a triangle with height 1 and base 2*δ* (see Eq. (8)). Thus, from Eq. (13), the following explicit expression originally proposed by Knutson [6] approximates the size distribution:

$$G(D_{p}(x=1))=\left[N(V)\left|\frac{C^{\prime }(D_{p})}{C(D_{p})}-\frac{1}{D_{p}}\right|\right]/\left[\delta p(D_{p}(x=1))\right].$$ (14)

The mobility for *x* = 1 corresponding to voltage *V* is computed from the following equation:

$$Z_{p}(x=1)=\frac{Q_{c}\ln(r_{2}/r_{1})}{2\pi VL}=Q_{c}/(2\pi V\Lambda ).$$ (15)

The value of *D_p_* corresponding to *Z_p_* is computed based on Eq. (2). A description of the iterative method used to solve this implicit equation for *D_p_* is given in Appendix A.

To assess the accuracy of using Eq. (14) in computing G(*D_p_*), a comparison will be made for the peak in the size distribution obtained using this approximate method versus the true peak in the size distribution.

#### 2.2.2 Approximation 2

A widely used calibration and measurement method [7] is to measure the peak voltage for the 100.7 nm SRM spheres and the unknown particles. From Eq. (15), the mobility of the unknown particle, $Z_{p}^{u}$, can be expressed as the mobility of the 100.7 nm particle, $Z_{p}^{SRM}$, multiplied by the ratio of the peak voltages,

$$Z_{p}^{u}(x=1)=Z_{p}^{SRM}(x=1)V_{p}^{SRM}/V_{p}^{u},$$ (16)

with the value of $Z_{p}^{SRM}$ computed from Eq. (2). Three assumptions are used in this approach. First, the peak in the mobility distribution for the SRM is assumed to be the mobility corresponding to the peak in the diameter distribution of the SRM. Secondly, the peak in the measured size distribution is assumed to be the diameter inferred from the peak in the voltage distribution. The third assumption is that the size distribution is symmetric about *x* = 1.

#### 2.2.3 Accuracy of Approximate Methods

Now let us compare the accuracy of the above approximate approaches for size distributions close to that of the 60 nm and 100 nm spheres. We assume size distributions that are Gaussian with peak diameters of 60.7 nm and 101.6 nm and with standard deviations of 4.3 nm and 2.6 nm. The number concentration is computed as a function of voltage using Eq. (12). We use this as “data” and compute the peak size using the two approximations described above. As seen in Table 1 for the 60.7 nm spheres, Approximation 1 is significantly more accurate with a difference of less than 0.01 % compared to a 0.71 % difference for Approximation 2. The smaller the ratio of the reduced standard deviation of the transfer function to that of the size distribution function, the better Approximation 1 should be. This is the case for the example in Table 1. For the 60 nm sphere the ratio, 0.08, is about three times smaller than that for the 100 nm sphere and the relative difference from the correct diameter is about five times smaller for the 60 nm spheres. However, in both cases the agreement with the correct value is within 0.05 %. Ehara *et al.* [3] have investigated the accuracy of Approximation 1 and 2 for the case of skewed Gaussian distributions with a peak particle size of 100 nm. For Approximation 1, the number mean diameter was accurately retrieved even for size distributions with widths small relative to the transfer function width. For Approximation 2, a significant deviation was observed for broad size distributions with the standard deviation/mean size > 0.05. This result is consistent with our analysis that both Approximations work well for the narrowly distributed 100 nm spheres, but that Approximation 2 does not work as well for the more broadly distributed 60 nm spheres.

## 3. Physical Properties: Expressions Used and Estimated Uncertainty

The relevant physical parameters are evident from looking at three key equations for making size measurements using the DMA. The first relates the electrical mobility to particle diameter, Eq. (2), and includes the electron charge, viscosity, and the slip correction, which in turn depends on the mean free path. The mobility at the peak in the transfer function [Eq. (6)) evaluated at *x* = 1] is a function of the classifier geometry including the inner and outer diameter and the length of the classifier region and of the sheath flow. The predicted particle concentration as a function of voltage depends on the bipolar charging function, *p*(*D_p_*) as seen from Eq. (12) and on the number concentration measurement.

### 3.1 Charge of an Electron − *e*

The magnitude of the charge on the electron and its combined uncertainty (1 standard deviation) is equal to (1.6021892 ± 0.0000046) × 10^−19^ C [8]. The standard relative uncertainty is about 3 × 10^−4^ % and is negligible in assessing the overall uncertainty in the particle diameter.

### 3.2 Viscosity − *η*

In 1945, Birge [9] reported the weighted average value of the viscosity of dry air at 23.00 °C as *η*_0_ = (1.83245 ± 0.00069) × 10^−5^ kg m^−1^ s^−1^ from six different results, correcting for temperature by using the Sutherland equation. This air viscosity value was used in the certification measurements of SRM® 1963 [2]. For consistency, we also consider the Birge result as the reference viscosity for this study. Once the reference viscosity at 23.00 °C is determined, the viscosity for other temperatures can be obtained using the Sutherland formula as discussed by Allen and Raabe [10],

$$\eta =\eta_{0}{\left(\frac{T}{T_{0}}\right)}^{1.5}\left(\frac{T_{0}+110.4\;K}{T+110.4\;K}\right),$$ (17)

where *T*_0_ is the absolute reference temperature (296.15 K) and *T* is the absolute temperature.

The value of the viscosity of dry air at 23.00 °C from Birge [6], has a 0.038 % relative standard uncertainty. The air flowing through the DMA has an estimated 7 % relative humidity. The decrease in the viscosity from the addition of water is estimated as 0.080 % based on the viscosity of water and its volume fraction of the air. This value of 0.080 % is taken as the standard relative uncertainty in the air viscosity resulting from the presence of water vapor. Computing the standard relative combined uncertainty of viscosity as the root-sum-of-squares, a value of 0.089 % is obtained. As is seen in Sec. 7, the uncertainty in the viscosity does not affect the particle diameter uncertainty when calibrating the DMA with a particle of known size.

### 3.3 Slip Correction − *C*(*D_p_*) and Mean Free Path − *λ*

The Cunningham slip correction factor, which corrects for noncontinuum gas behavior on the motion of small particles, is given by

$$C(D_{p})=1+K_{n}[A_{1}+A_{2}\exp(-A_{3}/K_{n}]=1+K_{n}A,$$ (18)

where the Knudsen number is twice the mean free path of air divided by the particle diameter (*K_n_* = 2*λ*/*D_p_*), *A*_1_, *A*_2_, and *A*_3_ are dimensionless constants, and *A* is termed the slip correction parameter. The quantity *A* is of key importance in the uncertainty analysis in Sec. 8.2. In our analysis two sets of values are used for the slip correction constants: *A*_1_ = 1.142, *A*_2_ = 0.558, and *A*_3_ = 0.999 (Allen *et al.*, 1985 [11]) and *A*_1_ = 1.165, *A*_2_ = 0.483, and *A*_3_ = 0.997 (Kim *et al.*, [12]). The first expression, which was used in the measurement of SRM^®^ 1963, was obtained using a Millikan apparatus with monosize polystyrene spheres having diameters of about 1 µm, 2 µm, and 4 µm. The second expression was obtained using reduced pressure measurements with a Nano-DMA^1^ on accurately sized calibration particles with diameter of 269 nm, 100.7 nm, and 19.90 nm [12]. Over the Knudsen number range from 1.35 to 2.25, which corresponds to a diameter range from 60 nm to 100 nm, the combined relative standard uncertainty in the Kim *et al.* [12] expression is 1.0 % or slightly less. The study by Allen *et al.* [11] does not contain an estimate of the combined relative standard uncertainty. Both of these sets of values will be used in computing the peak diameter.

The mean free path *λ* is needed to compute the slip correction. It cannot be directly measured, but instead is determined from the kinetic theory relationship for viscosity,

$$\eta =\phi \rho \bar{c}\lambda ,$$ (19)

where *ϕ* is a constant dependent upon the intermolecular potential, *ρ* is the gas density, and $\bar{c}$ is the mean velocity of gas molecules. The value of *ϕ* = 0.491 is derived by assuming hard elastic spheres with repulsive forces between the molecules [13]. The certification measurements of SRM^®^ 1963 [2] used *λ*_0_ = 67.3 nm for the mean free path of air at 101.325 kPa and 23.00 °C. Once the reference value of *λ*_0_ has been chosen, it can be corrected for any pressure and temperature using Willeke’s relation [14]

$$\lambda =\lambda_{0}\left(\frac{T}{T_{0}}\right)\left(\frac{P_{0}}{P}\right)\left(\frac{1+(110.4\;K)/T_{0}}{1+(110.4\;K)/T}\right),$$ (20)

where

*λ*_0_ = 67.3 nm, for air at *T*_0_, *P*_0_
*T*_0_ = reference temperature, 296.15 K
*P*_0_ = reference pressure, 101.33 kPa
*T* = air temperature inside the classifier
*P* = air pressure inside the classifier.

Equation (20) is used in the subsequent analysis for the mean free path of air.

### 3.4 Geometric Factors

The critical dimensions for the DMA are the inner radius *r*_1_ = 0.937 cm, the outer radius *r*_2_ = 1.958 cm, and the classifying length *L* = 44.44 cm. These dimensions were measured at NIST for the DMA used in this study. The quantity *Λ* defined in Eq. (7) has a value of 0.60299 cm. The uncertainty in this value is not needed, since the DMA is calibrated with a particle of known size. An error in either the geometric dimension or in the volumetric flow rate is corrected by this calibration as discussed in Sec. 2.2.

### 3.5 Flow Rate

The nominal sheath and aerosol flow rates are 20 L/min and 0.5 L/min. The actual sheath flow rate is calibrated before each size-distribution measurement. The average change in the flow between two successive calibrations is 0.1 %. The leakage rate in the recirculation system is measured to be 0.020 cm^3^/s. This corresponds to about 0.25 % of the 0.5 L/min aerosol flow [15].

### 3.6 Flow Ratio

The ratio of the aerosol flow to the sheath flow is 0.025 based on the nominal flows. The uncertainty in the flow ratio is + 2 %, − 7 %. The asymmetry arises from the corrected sheath flow being about 2 % larger than the nominal value of 20 L/min.

### 3.7 Charging Probability

The quantity *p*(*D_p_*), the probability that a particle of size *D_p_* has a single unit of positive charge, is needed both in computing the size distribution function in Eq. (14) and also in Eq. (11) for calibrating the DMA with a particle of known size. The following expression for *p*(*D_p_*), determined by Wiedensohler [16], is used in our analysis:

$$\log_{10}p(D_{p})=\sum_{i=0}^{5}a_{i}{\left(\log_{10}\frac{D_{p}}{D_{unit}}\right)}^{i},$$ (21)

where *D_unit_* = 1 nm, *a*_0_ = −2.3484, *a*_1_ = 0.6044, *a*_2_ = 0.4800, *a*_3_ = 0.0013, *a*_4_ = −0.1553 and *a*_5_ = 0.0320. This approximate expression is within 3 % of the value predicted by Fuch’s theory [17] for particle sizes of 50 nm, 70 nm, and 100 nm: The reduced difference, [*p*(approximate)—*p*(Fuchs)]/*p*(Fuchs), equals −2.6 % for 50 nm, 0.7 % for 70 nm, 1.6 % for 100 nm.

## 4. Particle Sizing Facility

The equipment comprising the DMA measurement system consists of five components: the aerosol generator, the electrostatic classifier, the condensation particle counter, the recirculation system, and the data acquisition system. All of the components, except the recirculation system and a modified aerosol/sheath inlet, are commercially available equipment. The equipment and methodology used here are similar to that used previously in performing particle calibration measurements [18].

### 4.1 Aerosol Generation

Two types of aerosol generators were used. A pneumatic nebulizer was used for generating the new 100 nm spheres and an electrospray generator was used for the 60 nm spheres.

#### 4.1.1 Pneumatic Generator

The 100 nm polystyrene sphere aerosol is generated using an Aeromaster Constant Number PSL Standard Particle Generator manufactured by JSR Corporation. The generator is a pneumatic atomizer that operates by using filtered compressed (100 kPa gauge) air to generate a high velocity gas jet, which, in turn, creates a low pressure region near the tube exit. The pressure imbalance acts to draw liquid with suspended PSL spheres into this region. The high air velocity breaks up the liquid into a wide range of droplet sizes with large droplets impinging on the wall and dripping into a container, and smaller droplets flowing with the air stream. Fresh liquid is constantly drawn into the spray region resulting in a steadier particle concentration than would occur with dripping of the impinging drops back into the liquid reservoir. Another feature that improves the generator’s stability is a temperature-controlled capillary feed line.

The aerosol passes through a heated tube where the liquid evaporates leaving only the solid PSL particles as an aerosol. The flow then enters a diluter where it joins a clean air stream. The flow passes through a bipolar charger to reduce the droplet charge. The aerosol flow of about 14 L/min then enters an integrating chamber. The chamber has a volume of approximately 14 L and serves to dampen any short term fluctuation in the aerosol concentration. This generator maintains a steady particle concentration typically within 2 % during a fifteen minute voltage scan.

The charged flow leaves the chamber and reaches the classifier via a path containing regulating vents. The vents are adjusted to set the desired flow rate entering the classifier, while the remaining flow is sent to the exhaust. This is necessary since the aerosol generator supplies a flow of approximately 16 L/min (267 cm^3^/s) while the typical flow into the classifier is less than 2 L/min (33 cm^3^/s).

#### 4.1.2 Electrospray Generator

**Fig. 2:**
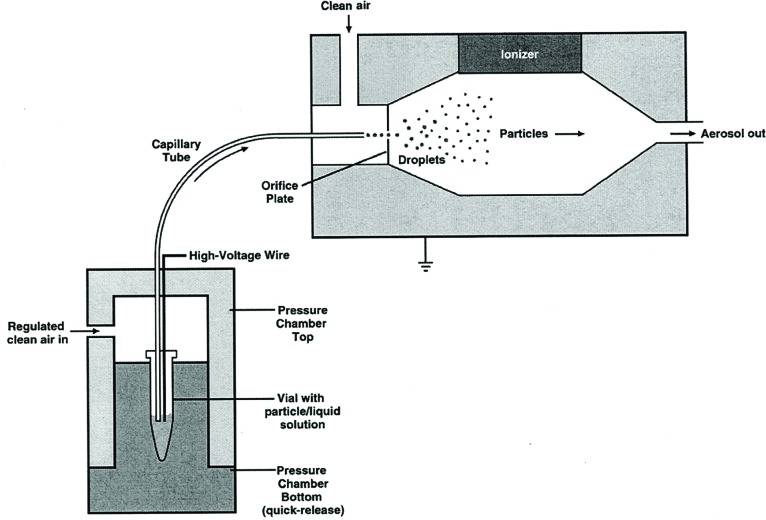
Schematic diagram of the electrospray adapted from the TSI 3480 technical manual.

The electrospray system, TSI Model 3480 illustrated in Fig. 2, generates a constant output aerosol at positive pressure. Several modifications to the standard operation protocol were used to enhance the electrospray performance for the PSL suspensions. When operating the electrospray to aerosolize PSL particles, the small diameter capillary would periodically clog by the PSL suspension. To minimize the clogging problem, the electrospray was operated with a 40 µm I.D. silica capillary rather than the standard 25 µm I.D. capillary. The increased tube diameter results in a nominal liquid feed rate of 1 × 10^−11^ m^3^/s (0.6 µL/min) for a chamber overpressure of about 25 kPa (1/4 atmosphere). This larger feed rate, by approximately 10 times, resulted in the electrospray operating in a pulsating mode rather than the cone-jet mode. In the pulsating mode [19], the liquid emerging from the capillary oscillates between the dripping and cone-jet modes. Stable and continuous operation without clogging can be run for an extended time period. The droplet size distribution is broadened but this does not affect the dispersed PSL particle size. We found the pulsating mode to be useful for dispersing PSL spheres larger than 50 nm.

The mean droplet size with the electrospray is smaller than that produced by pneumatic nebulization resulting in less residue from the nonvolatile impurities from the water/PSL sample material on the surface of the PSL sphere after the water has evaporated. A study by Mulholland *et al*. [20] found that the PSL sphere diameter obtained using electrospray was about 1 % smaller than that obtained by pneumatic nebulization for 100 nm spheres and about 5 % smaller for 50 nm spheres. This study was carried out with a prototype electrospray with a smaller capillary diameter, 25 µm, and for a different pneumatic nebulizer, which is thought to produce a larger mean droplet size than that produced by Aeromaster. Another set of measurements was made using the Aeromaster and the current electrospray with a 25 µm capillary [7]. The PSL diameter generated with the electrospray was 2.5 % smaller for 55 nm spheres and 3.9 % smaller with 64 nm sphere. The uncertainty in these differences is about ± 1.3 %; that is, 3.9 % ± 1.3 % and 2.5 % ± 1.3 %. During a set of screening measurements made the month after the 100 nm certification measurements, the peak particle size was measured for nominal 64 nm spheres using both the Aeromaster and the current configuration of the electrospray with the 40 µm diameter capillary. It was found that the PSL sphere size obtained with the electrospray was about 3 % to 4 % smaller than the value obtained with the pneumatic nebulizer. While there were only two repeats using each generator, the result is consistent with the previous studies [20, 7] and is a strong indication that there is less of a residue effect with electrospray compared to the pneumatic atomizer. Additional measurements of the residue layer are presented in Sec. 8.3.8.

A flow of 33.3 cm^3^/s (2 L/min) of dry-filtered air enters the spray system. A co-flow of CO_2_, which is commonly used to prevent corona discharge, was not used in our experiments because of the difficulty in accurately determining the viscosity of such a gas mixture. As the particles flow through the orifice (See Fig. 2) they are exposed to a bi-polar distribution of ions produced by 370 MBq Po^210^, which produces α radiation. The particles acquire a Boltzmann charge distribution described by Eq. (21).

The sample is introduced into the cell, pressure applied, and the air flow set. The voltage is increased, and the droplets are observed through the viewing window illuminated by a light emitting diode. The voltage is increased until the current output displayed by the generator varies by less than 2 nA over the nominal 10 min required for measuring the size distribution. Typically the voltage was about 1.5 kV, the current was about 120 nA, and the spray pattern appeared as a pulsating tip. The voltage range used in these tests is lower than typically used with the electrospray. This is in part a result of not using the CO_2_ gas, which resulted in an onset of corona breakdown at a lower voltage. In some cases the voltage was increased to 1.8 kV or 2.2 kV to obtain stable behavior. The resulting peak number concentrations were in the range of (200−450) particles per cm^3^. The particle number concentration for a fixed DMA voltage was observed to drift by as much as 10 % over a measurement period. Measurements were made at a voltage close to the peak voltage at the start of the experiment, at the middle, and at the end to estimate a linear drift in the concentration over the course of the experiment. All the number concentration data were adjusted with the linear fit to give the estimated concentration at the time of the peak measurement. These adjustments were made for both the SRM calibration measurements used to find the corrected flow and for the 60 nm sphere measurements.

Once started, the system generally provides a steady output of particles. Over a five day period during which the electrospray was run on the order of four hours a day, one capillary had to be replaced because of clogging. The filtering of the suspension of particles in the electrolyte with a 1.2 µm pore size filter may have helped minimized the particle clogging. The other standard precaution of running particle free electrolyte for one hour in the dripping mode after the test to clean particles away from the tip was used. Also, a 1 L accumulator was used directly after the generator to reduce concentration fluctuations.

### 4.2 DMA

The DMA used in these experiments is a model 3071A Electrostatic Classifier manufactured by TSI Incorporated. The DMA separates aerosol particles based on their electrical mobility as described in Sec. 2. This allows for a flow of monosize particles to exit the classifier. The DMA measurement system contains a bipolar charger (TSI Model 3077 Aerosol Neutralizer) consisting of 7.4 × 10^7^ Bq (2 mCi) of Kr 85 radioactive gas contained in a capillary tube. Here the particles collide with bipolar ions resulting in an equilibrium charge distribution that is a function of the particle size. The probability of a sphere having a single charge is given above by Eq. (21). For example, 100 nm particles would emerge from the bipolar charger with 42 % of the particles uncharged, 21 % with a +1 charge, 27 % with a −1 charge, and smaller percentages with multiply charged particles. The charger is removed when using the electrospray, since it already contains a bipolar charger.

After passing through the bipolar charger, the aerosol flows to the DMA. As shown in Fig. 1, the DMA is a long cylindrical chamber with a central rod concentric to the walls of the chamber so that an annulus is formed between the rod and the chamber walls. The rod voltage can be adjusted from about −10 V to −10 000 V. The outer cylindrical chamber is kept at ground voltage, allowing for an electrical field to develop within the annulus. The aerosol flow enters the top of the chamber and is joined by a sheath flow of clean air. Both flows travel through the annulus to the bottom of the chamber. Along the way, positively charged particles move toward the center rod due to the rod voltage. A small slit in the rod allows for the passage of particles with electrical mobility with an approximate mean mobility, *Z*(*x* = 1), computed by Eq.(15). The flow leaving the DMA, now comprised of nearly monosize particles, passes through the rod opening, exits the classifier, and then flows into the condensation particle counter where the number of particles is counted. The rest of the flow leaves the classifier through an excess flow outlet and enters the recirculation system.

An improvement was made on the DMA [21] to allow higher resolution measurements of the particle size distribution. Based on field model calculations, a modified aerosol/sheath inlet was designed and evaluated. The new design allows the aerosol/sheath flow ratio to operate as low as 0.015. It was applied to the measurement of the NIST 0.1 µm SRM^®^ 1963 and it was found that the improved DMA can accurately measure the standard deviation of this narrowly distributed aerosol with a ratio of the standard deviation to peak particle size equal to about 0.02.

### 4.3 Recirculation System

A recirculation system pumps the sheath air through the classifier, draws out the excess air and then conditions it before returning it as the sheath air flow. Recirculating the excess air into the sheath inlet ensures that the excess and sheath flow rates are equal to within 0.01 %. This significantly reduces uncertainties in the size calculations that would be present if these flows were not matched and needed to be measured independently. The recirculation system was not supplied with the TSI model 3071A Electrostatic Classifier, but was built separately at NIST for use with the electrostatic classifier.

**Fig. 3:**
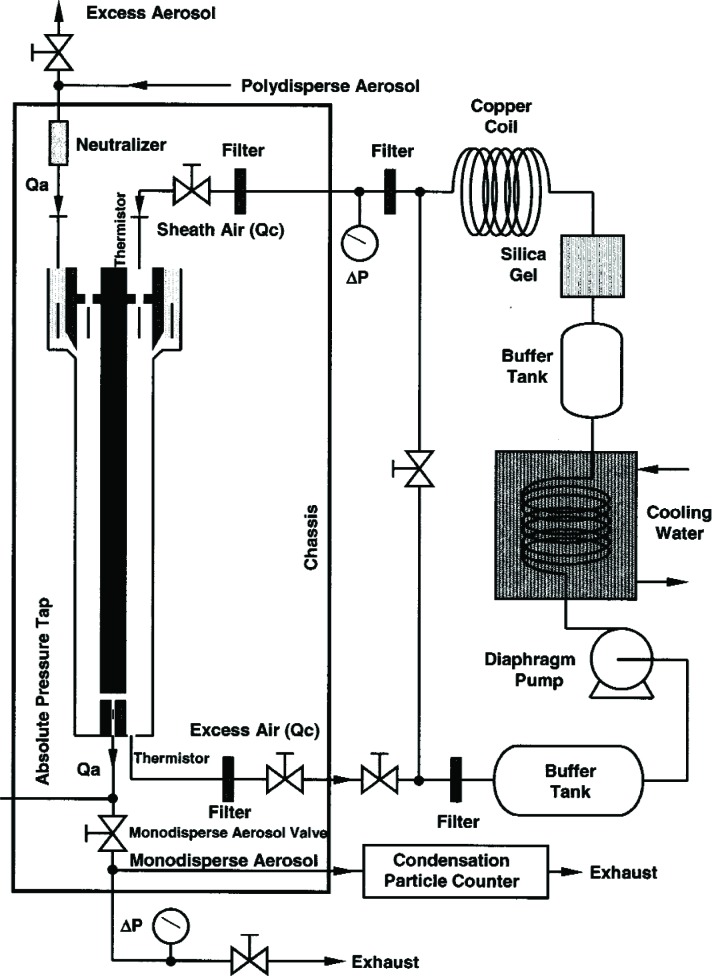
Schematic of the flow through the DMA and the recirculation flow from the excess flow outlet back to the sheath air inlet. The location of the thermistors near the sheath inlet and excess outlet are shown along with the point at which the pressure is monitored. The valve shown just downstream of the pressure monitoring point is actually within the DMA instrument chassis.

A schematic diagram of the recirculation system is included in Fig. 3. Excess air leaves the classifier, passes through an adjustable needle valve and is then filtered through an ultra high efficiency pleated membrane cartridge filter to remove any particles. From the filter, the flow enters a buffer tank. The tank is a brass cylinder 40 cm long with a volume of 6 L and serves to dampen the pulsations caused by the pump. After the buffer tank are two diaphragm pumps connected in parallel. The operator can choose to have only one pump operate for low flow rates, or have two pumps operate for higher flow rates. After the pumps, the flow travels through coils submerged in a water bath. Since the pumps heat the flow, the water bath is needed to reduce the temperature of the flow. The bath itself is cooled by a second set of coils carrying tap water. After leaving the bath, the flow enters another buffer tank, the same size as the first one, to further dampen the effects of the pump. From there the flow travels through a drier packed with silica gel to remove moisture from the flow. It then travels through a coiled section that acts as a heat exchanger to allow the flow to reach room temperature. The conditioned flow is then split into two flows. Most of the flow is sent through another pleated membrane cartridge filter, to remove any residual particles, and then into the top of the classifier as the sheath flow. A small portion of the flow is diverted back into the recirculation system, joining the excess air as it leaves the classifier. Adjusting the by-pass needle valve provides a high resolution adjustment of the sheath flow. A key design feature is that the sheath flow equals the excess flow.

The second key feature is that the buffer tanks together with the filters and the drier greatly reduce the pressure fluctuations from the diaphragm pumps. Pressure fluctuations could be a problem because they would cause mixing fluctuations, which, in turn, would affect the resolution of the size distribution measurement. A 1.33 kPa (10 torr) differential pressure transducer with a 1 ms time response was used to measure the pressure fluctuations in the excess flow line just outside the DMA chassis (Fig. 3). The pressure was recorded versus time for the DMA operated at a nominal flow of 20 L/min with the pump and also recorded for a steady flow of 20 L/min of nitrogen from a gas cylinder through the DMA with the pump and recirculation system disconnected. Pressure measurements were also made just downstream of the pump. The frequency of the pump pressure pulsations was about 29 Hz and the amplitude exceeded 1333 Pa (10 torr). There was a small amplitude peak for the buffered flow at about 29 Hz. The amplitude was at least 270 times smaller than the pump amplitude. Some periodicity was also observed for the steady flow case. The standard deviations of the pressure for the buffered flow was about 5.1 Pa compared to about 3.7 Pa for the cylinder flow (cylinder). From a power spectral analysis (see Appendix B) it was found that all three cases have peaks near 30 Hz and 60 Hz though much reduced in magnitude for the recirculations flow and cylinder flow.

There is also indirect evidence that the flow fluctuations were small. Flow fluctuations could cause mixing of the aerosol flow with the sheath flow. This would lead to a broadening of the size distribution. The narrowness of the size distribution of the SRM^®^ 1963 spheres (Fig. 5) described in Sec. 6.1 is evidence that the pulsations from the pump are not affecting the resolution of the size distribution measurements.

### 4.4 Support Measurements of Pressure, Temperature, and Voltage

In addition to the recirculation system, equipment was added to the DMA measurement system to obtain accurate pressure and temperature measurements. The barometric pressure is measured near the monodisperse exit (see Fig. 3) using a Mensor Corporation Model 4011 digital pressure transducer containing an ion implanted silicon strain gage. Thermistors provide accurate temperature measurements at two locations in the sheath flow: one is located in the upper sheath flow just before it enters the DMA and the other is located after the DMA exit. The thermistors are type CSP Thermoprobes manufactured by Thermometrics Incorporated, with NIST traceable calibrations. The pressure transducer and each of the thermistors provide an updated digital output to the data acquisition system at a rate of 1 Hz for continual monitoring of the environmental conditions used in the diameter calculations.

The appropriate pressure for computing the slip correction in Eq. (19) is the pressure within the DMA; thus the pressure drop across the exit slit must be determined to correct the pressure reading made at the monodisperse outlet tube. To keep the pressure drop as small as possible, the monodisperse aerosol valve was always fully open. The pressure drop inside the classifier was measured as a function of sheath flow as well as aerosol flow through the classifier in a separate set of measurements [7]. Pressure measurements with varied flows determined that the pressure difference was a function of the aerosol flow rate only. Results for the pressure drop measurements for a sheath flow of 20 L/min are reported in Table 2.

The DMA voltage affects the measurement of the unknown particle size directly and also indirectly through the calibration measurement of the 100 nm SRM^®^ 1963. Uncertainties in the voltage will affect the calculated particle mobility as given in Eq. (2), which will in turn affect the measured particle diameter. A high voltage (1000 V to 10 000 V) calibration facility was set up to measure the voltage of the DMA rod using a high voltage divider and a digital voltmeter. A Spellman HUD-100-1 precision resistor ladder was used to step down the rod voltage. The output signal was then measured using a Fluke Corporation 8060A digital multimeter. It was critical that both the resistor ladder and the volt meter be operated in a high impedance mode to obtain an accurate voltage. Both the resistor ladder and the multimeter have relative standard uncertainties of 0.05 % of the nominal reading over the measurement range. The resistor loop provides DMA rod voltage measurements with a relative combined standard uncertainty of ± 0.08 %.

### 4.5 Particle Concentration

The particle concentration is determined using a model 3022A Condensation Particle Counter (CPC) manufactured by TSI Incorporated. The CPC detects particles by condensing supersaturated butanol vapor onto the particles to increase their size before they enter the optical sensing zone where they are counted. The CPC is capable of detecting particles of size 7 nm and larger. The nominal number concentration is 100 cm^−3^ to 200 cm^−3^ for the 100 nm spheres produced by the pneumatic nebulizer and 200 cm^−3^ to 450 cm^−3^ for the electrospray.

### 4.6 Data Acquisition

The data acquisition system consists of a desktop computer equipped with data acquisition boards and software for communication with the instruments. Communication with the CPC and the electrostatic classifier is accomplished via an RS-232 serial communications port. Information from the digital pressure transducer is collected using a National Instruments PCI 6503 digital input/output board. Thermistors are connected to a National Instruments TBX 68T Isothermal Terminal Block, which relays the temperature information to a National Instruments 4351 PCI board. A customized data acquisition software program is used to control the voltage setting of the DMA and sequence through a series of voltages with a fixed time interval for each voltage. The number concentration, pressure, two thermistors, and voltage are recorded every second. Another custom program averages the data and provides the averages and standard deviations for the quantities above for each voltage setting. Both programs are written using National Instruments LabVIEW software.

## 5. Particle Characteristics and Sample Preparation

The 100 nm spheres were synthesized by Duke Scientific Corporation using emulsion polymerization with styrene monomer. The bulk suspension was diluted with 18 MΩ deionized water filtered with a 0.04 (µm) pore size filter. The sample mass fraction is 0.5 % and the volume per sample is 5 mL. The mass fraction of surfactant is 0.021 % and consisted of sodium 1-dodecanesulfonate and 1-dodecanol. There is also 0.006 % electrolyte remaining from the synthesis. There is no added preservative.

To prepare samples for analysis by the DMA, five drops of the suspension were diluted with 200 cm^3^ of deionized water filtered with an 0.2 µm pore size filter. The 100 nm SRM® 1963 samples were also prepared by diluting five drops of the SRM with 200 cm^3^ of deionized, filtered water. The nominal droplet size for both of these samples and for the 60 nm spheres was about 0.040 cm^−3^.

The 60 nm spheres were synthesized by JSR Company using emulsion polymerization with styrene monomer. In this case the surfactant is synthesized into the polymer itself as carboxyl groups so that no additional surfactant is added.

One drop of the 60 nm PS spheres is added to the 1 cm^3^ capsule filled with ammonium acetate solution with a conductivity of about 0.2 S/m (1 S ≡ 1/Ω). This corresponds to about a 20 mmol/L solution. The ammonium acetate sublimates so that it does not contribute to a residue layer. The electrolyte/particles suspension was filtered with a 1.2 µm pore size filter to remove large particles such as dust. This was done to minimize particle clogging of the electrospray capillary.

## 6. DMA Measurement Process

Two types of measurements were taken with the DMA. One measurement was the accurate determination of the peak in the size distribution for the 60 nm or 100 nm spheres and involved two steps. The first step was the calibration of the DMA using SRM® 1963. The second step was the actual determination of the peak particle size using the DMA. The second DMA measurement involved determining the peak voltage for a number of different sample bottles to test for homogeneity of the samples. The method for determining the peak voltage is discussed in this section, but the experimental design and the analysis of variance to verify the homogeneity of the different samples is presented in Sec. 7.

### 6.1 100 nm Spheres and SRM® 1963

The particle suspension was prepared as described above and the pneumatic aerosol generator was operated at an aerosol flow of about 16 L/min with a 14 L accumulator. The sheath flow was set to 20 L/min with the DMA excess air valve fully open by adjustment of the two valves in the recirculation system. The aerosol flow was set to 0.5 L/min with the monodisperse valve fully open by adjusting the bypass valves controlling the aerosol inlet flow. As discussed previously, this approach minimizes the pressure drop between the DMA column and the outlet flow. A preliminary scan of number concentration versus voltage was taken to determine the peak voltage and voltages corresponding to a decrease in the number concentration by about 30 %, both above and below the peak. This corresponded to a voltage range of about 300 V for the 100 nm spheres and SRM® 1963, with peak voltage of about 3500 V. These values allowed for 16 voltage channels, spaced by 20 V each, to be used in the data acquisition process. The data acquisition was designed to collect number concentration once a second for 45 s at each voltage. Data was also collected every second for the pressure in the DMA and for the temperature of the DMA inlet and exhaust gas. It was found that the CPC reached a steady concentration within about 20 s of a voltage change. The average number concentration, average pressure, and average inlet and outlet temperature were computed over the final 20 s of the 45 s dwell time at a fixed voltage. The inlet and outlet temperatures typically differed by about 0.25 °C. The DMA temperature was taken to be the numeric average of the time averages for the inlet and outlet temperatures.

**Fig. 4:**
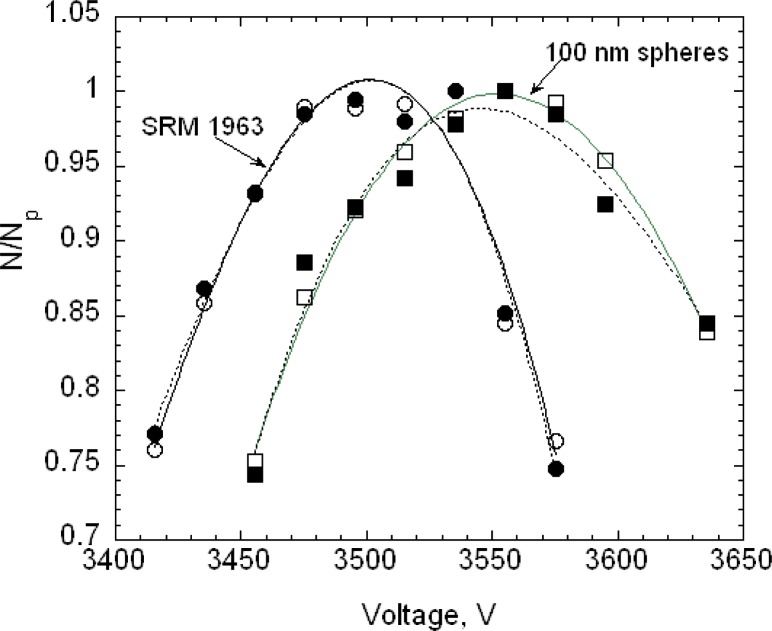
The number concentration normalized by the concentration at the peak is plotted versus voltage for two repeat measurements for the 100 nm spheres and for SRM^®^1963, 100.7 nm spheres used to calibrate the DMA. SRM run c (solid circle), g (open circle); 100 nm run b (solid square), d (open square) taken on 20 September 2004. Curves denote best fit cubics for the individual measurements (dashed curve fit for solid symbols; solid curve for open symbols).

For the 100.7 nm SRM® 1963 particles, 16 voltage-steps were taken with 8 or 9 of these steps used in determining the peak voltage. The voltage-steps used to determine the peak had typically the range *N*/*N_peak_* > 0.75, where *N_peak_* is the highest number concentration recorded by the CPC during a voltage scan. Two repeat data sets, taken on 20 September 2004, are shown in Fig. 4 along with the best fit cubic curve for each measurement series. The best fit peaks are 3501.2 V and 3502.0 V. If two more data points are added extending the range to *N/N_peak_* > 0.65, the peak voltages decrease slightly to 3500.5 V and 3498.5 V.

The best fit cubic curve for the data is obtained using the proprietary KaleidaGraph nonlinear least squares software. The peak in the curve is obtained by setting the derivative of the cubic equation to zero. It was found that five significant figures in the coefficients of the cubic resulted in a numerical uncertainty of less than about ± 0.04 % for the voltage and particle size peaks obtained in this study.

Two repeat data sets for the 100 nm spheres are also shown in Fig. 4. It is seen that the 100 nm spheres have a slightly larger peak voltage and distribution width. On each of three days, three voltage scans were made on the 100 nm spheres and four on the SRM® 1963 particles.

As was discussed in Sec. 2.2, the measured peak in the voltage for SRM® 1963 was compared with the predicted voltage, using the known peak size of 100.7 nm, the standard deviation of 2.0 nm and the transfer function integral to compute the predicted peak voltage. The flow rate was then adjusted from 20 L/min to this value times the ratio of the voltages to get the calibrated flow. For these two cases the corrected flows were 21.153 L/min and 21.149 L/min.

**Fig. 5:**
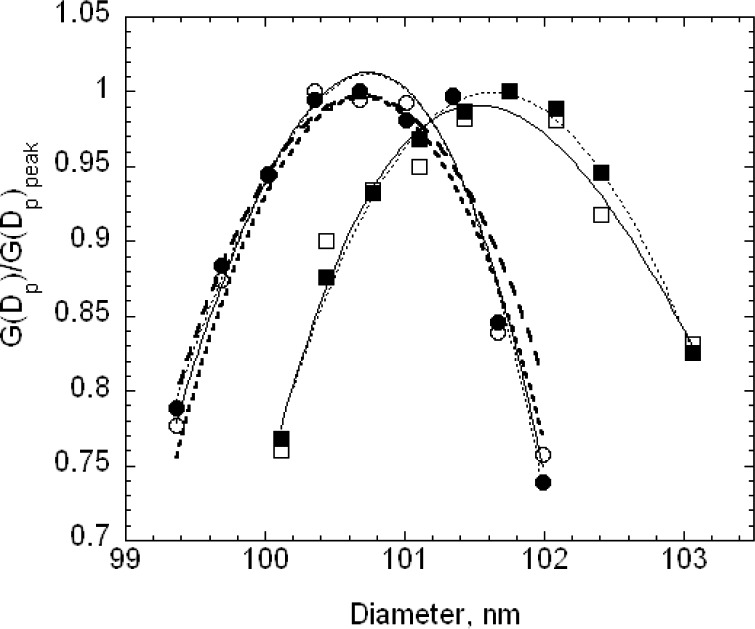
The number size distribution normalized by the peak in the distribution is plotted versus diameter for the 100 nm spheres and for the SRM^®^1963 spheres for the same data as Fig. 4. The short and long dashed curve corresponds to the Gaussian size distribution of SRM^®^ 1963 with the certified number mean diameter of 100.7 nm and standard deviation of 1.8 nm (narrower curve) and 2.0 nm.

The estimated size distribution, *G*(*D_p_*), based on the first approximation method, is obtained from *N*(*V*) using Eq. (14). To obtain the diameter *D_p_* corresponding to the voltage *V* requires two steps. First, the mobility *Z_p_* corresponding to the voltage is computed using Eq. (22) with the corrected flow rate. Then the diameter corresponding to the mobility is computed iteratively using Eq. (2) together with the expressions for the slip correction, Eq. (18), and the expression for the mean free path, Eq. (20). The size distributions corresponding to the plots of *N*(*V*) in Fig. 4 are plotted in Fig. 5. The peak diameters based on the cubic fits are 101.53 nm and 101.63 nm. In the certification measurements, the 100.1 nm diameter point was not included and the corresponding peak diameters were 101.70 nm and 101.68 nm. This represents a worst case in terms of fitting because of the asymmetry in *G*(*D_p_*) between the smallest and largest diameters in the fitting range, whether or not the point at 100.1 nm is included. However, even in this worst case, the difference in the two estimates is 0.17 % in one case and 0.07 % in the other.

The peak sizes of 100.73 nm and 100.71 nm for SRM® 1963 demonstrate the consistency of the calibration procedure, which adjusted the value of the sheath flow so that the measured peak in the voltage plot would equal the value predicted assuming a Gaussian size distribution with a peak at 100.7 nm. Figure 5 also provides a comparison of the measured size distribution and Gaussian distributions with a number mean size of 100.7 nm and standard deviations of 2.0 nm and of 1.8 nm. The agreement appears to be slightly better for the narrower Gaussian distribution. The value of the standard deviation obtained by transmission electron microscopy was 2.0 nm.

**Fig. 6:**
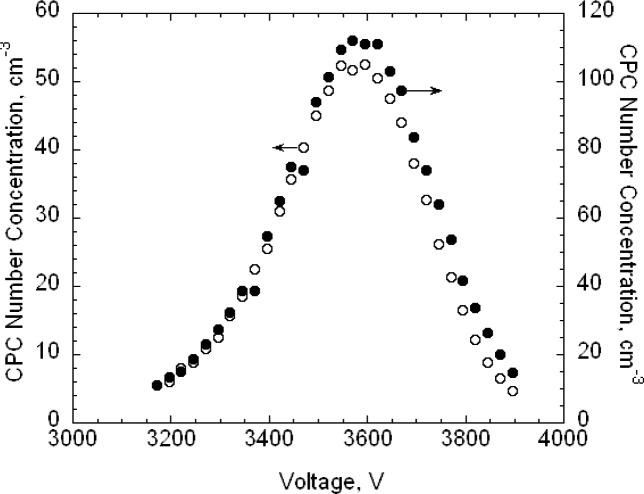
Plot of number concentration versus voltage for the full range of the size distribution. Displayed data is for 100 nm run d (open circle, left axis) with 1 drop per 200 cm^3^ and run f (solid circle, right axis) with 6 drops per 200 cm^3^. Data were taken on 14 October 2004.

The 100 nm spheres were also measured over wider size ranges to better define the full distribution and to see if the multiply charged dimers or trimers were interfering in the size measurement. A voltage scan from about 3100 V to 3900 V showed an almost 10 fold variation in the concentration as shown in Fig. 6. The results are plotted for both 1 drop and 6 drops of the particle suspension diluted in 200 mL of particle free deionized water. The six-fold increase in particles resulted in only about a doubling of the concentration. There appear to be two outlier data points around 3350 V and 3450 V in run F (Fig. 6), which might have resulted from the voltage failing to increase at the proper time. Subsequent observations made while observing the DMA display along with the computer display demonstrated that this would happen at infrequent intervals, perhaps once every two or three scans.

**Fig. 7:**
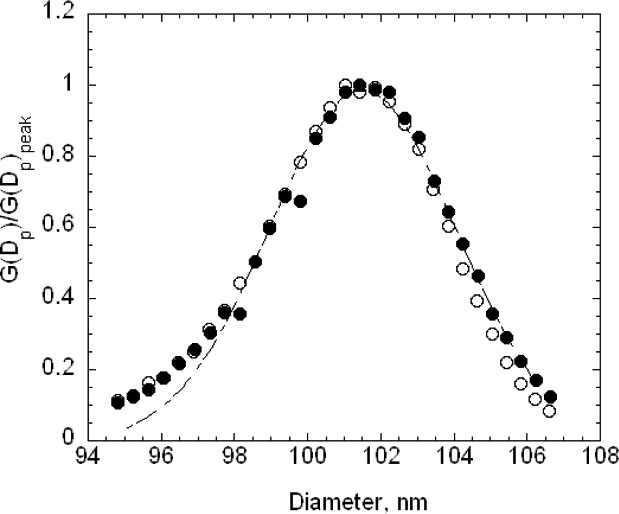
The normalized number size distribution is plotted for the same data set as Fig. 6. For comparison a Gaussian distribution with a mean 101.5 nm and a standard deviation of 2.5 nm is plotted as a dashed curve.

The reduced number distributions for the 100 nm particles, plotted in Fig. 7, agree well up to the peak. For particle diameters beyond the peak, a slight off-set in the distributions is apparent for particle diameters that increases to about 0.3 nm for the largest sizes measured. For comparison purposes, a Gaussian size distribution with a peak size of 101.50 nm and a standard deviation of 2.5 nm is also plotted. The Gaussian overlaps the two data sets for the reduced *G*(*D_p_*) > 0.4. For *G*(*D_p_*) < 0.4, the measured values are asymmetric at the smaller size particles.

**Fig. 8:**
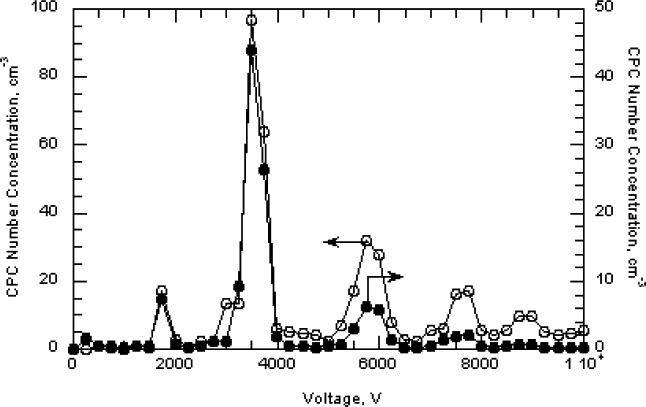
Number concentration versus voltage over the full voltage range of the DMA. The plot indicates the formation of dimers, trimers, and tetramers as well as the generation of doubly charged monomers. Data are for 100 nm run A, six drops / 200 cm^3^ (open circles, left axis) and run B, 1 drop/200 cm^3^ (solid circles, right axis). The voltage apparently failed to advance during three consecutive intervals starting at 4000 V and the number concentration was adjusted close to zero to be consistent with the run B as well as other data with the 100 nm spheres.

Two scans were made in 250 V increments from essentially zero to 10 000 V for the two particle concentrations used previously. As seen in Fig. 8, there is a prominent peak from the singly charged 100 nm spheres and a number of other peaks resulting from doubly charged spheres and from sphere aggregates including dimers, trimers, and tetramers. We adopt the terminology of mass spectrometry, in which the singlets, doublets, …multiplets always refer to the charge, and monomer, dimer, …multimer always refer to the number of primary particles in an aggregate. Then a singly charged dimer and a doubly charged trimer become a singlet dimer and a doublet trimer. There is a possibility that multiplet multimers will have mobilities overlapping that of the singlet monomer. The typical analysis region for sizing the 100 nm spheres was from 3450 V to 3650 V. If there were singlet trimers with voltage in the range of 6900 V to 7300 V, then the corresponding doublet trimers would range from 3450 V to 3650 V. For run A the particle concentration measured at 6990 V, 7240 V, and 7490 were (5.8, 6.0, and 16.5) cm^−3^, respectively, compared to a peak concentration of 97 cm^−3^. The possible impact of doublet trimers on the sizing of the 100 nm spheres will be discussed in Sec. 8.3.7. Over the voltage range corresponding to the full width of the 100 nm size distribution shown in Fig. 7, 3170 V to 3900 V, there would be a slight contribution from the doublet dimer in the small particle size region and from the doublet trimer in the peak particle size region.

### 6.2 60 nm Spheres

For the 60 nm spheres, the electrospray generator was used with a 2 L/min output followed by a 1 L accumulator. The measurement approach was similar to that used for the 100 nm spheres except that two 45 s scans were made at the start of the experiment at the peak voltage and then again at the end of the experiment. These additional measurements were to correct for instrument drift over the 15 min period of data collection. Previous diagnostic measurements indicated that the drift was much larger in this case than for the pneumatic nebulizer. Also, after finding evidence of an infrequent failure of the DMA voltage to be changed at the proper time, the correct voltage was verified/corrected at each time increment for the 60 nm spheres.

**Fig. 9:**
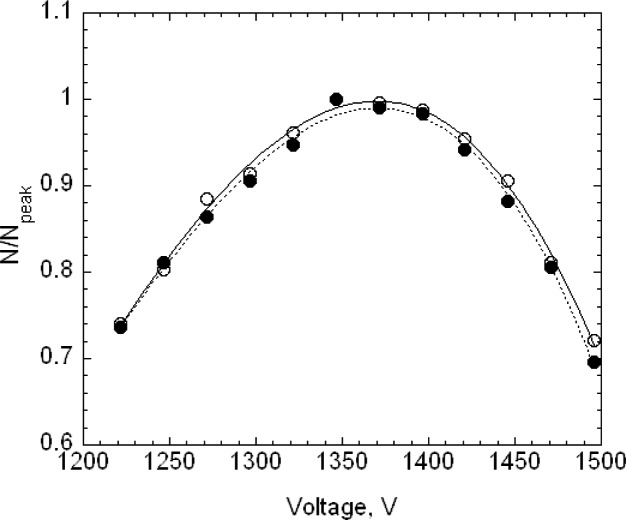
The number concentration normalized by the concentration at the peak is plotted versus voltage for two repeat measurements of the 60 nm spheres. Data are for runs g (open circles) and r (closed circles), taken on 16 February 2005. Displayed curves are for best cubic fits to the individual measurements (dashed curve fit for solid circles; solid curve for open circles).

The nominal peak voltage for the 60 nm spheres was 1370 V, with the voltage range extended over 350 V in 15 steps of 25 V each. The value of N/Np was about 0.6 at the largest and smallest voltage. The relatively large steps were required because of the broadness of the size distribution and the lower stability of the generator compared to the pneumatic generator. In this case, about 12 data points were fitted over the range of *N*/*N_p_* > 0.75 to determine the peak voltage. The results and cubic fits for runs F and G, taken on 16 February 2005, are shown in Fig. 9.

**Fig. 10:**
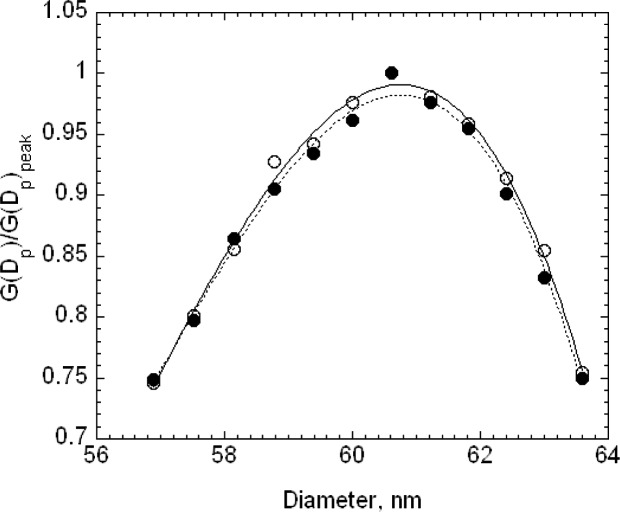
The number size distribution normalized by the peak in the distribution is plotted versus diameter for the same two 60 nm data sets as in Fig. 9.

The electrospray was also used with the SRM® 1963 spheres for the calibration of the DMA. In this case, 25 V increments were chosen rather than the 20 V increment used with the pneumatic generator. Otherwise the calibration was identical to the procedure described above. The results for the size distribution *G*(*D_p_*) are plotted in Fig. 10. The best fit peak diameters over the range *N*/*N_p_* > 0.75 are 60.62 nm for run F and 60.52 nm for G. Removing the lowest two points so that the fit is over the range greater than 0.80 results in a 0.03 nm decrease in the peak diameter for F and a 0.06 nm increase for G.

**Fig. 11:**
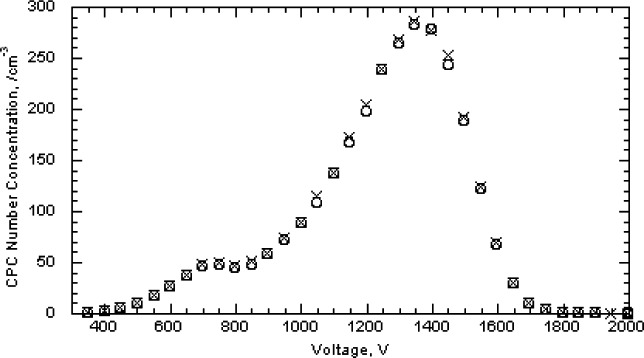
Number concentration versus voltage over the full size distribution of the 60 nm spheres. Data are for runs b (open circles) and c (x’s), taken on 25 February 2005.

**Fig. 12:**
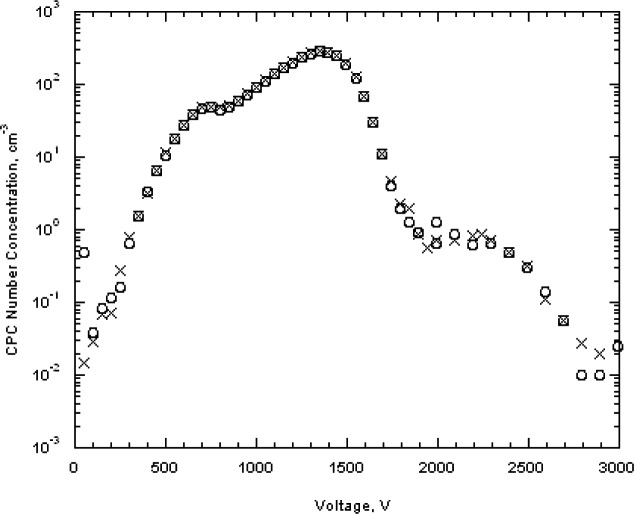
Data from Fig. 11 plotted using a logarithmic scale to show the much lower dimer concentration compared to the pneumatic aerosol generator.

As was done for the 100 nm particles, data were also collected over a larger range in voltage to obtain the full size distribution. The results, plotted in Fig. 11, indicate a plateau in the low voltage region. The doublet monomer would be classified at a voltage of about 675 V, which overlaps with the plateau in the small particle size of the size distribution. Based on the relative charging probability of a doublet monomer to a singlet monomer [16], the estimated particle number concentration of 18 cm^−3^, of the total of 48 particles cm^−3^, would be doublet monomers. A logarithmic plot of the data over a wider range is needed to depict the very low contribution of dimers and trimers and is shown in Fig. 12. The singlet dimer voltage is about 2200 V corresponding to the plateau region in Fig. 12. The ratio of singlet monomers to singlet dimers is approximately 3 × 10^−3^. So in this case, the contribution from multimers can be ignored.

**Fig. 13:**
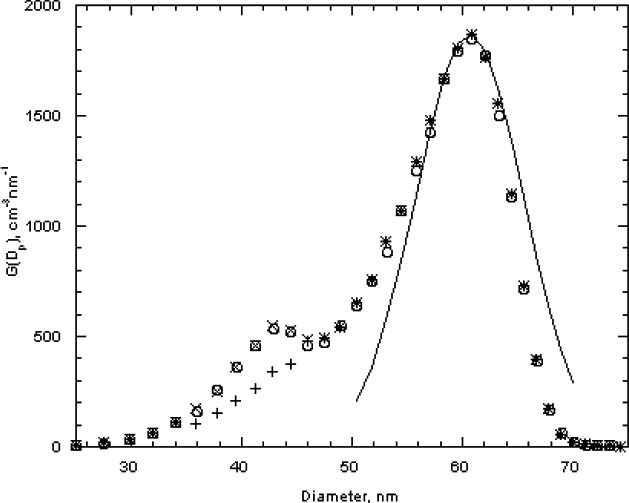
Number size distribution versus diameter based on the data from Fig. 11. The data is compared to a Gaussian with mean of 60.5 nm and standard deviation of 4.9 nm (solid curve). The cross symbols (+) denote data from run c, corrected for the contribution from doublet monomers (doubly charged monomer).

The number distribution *G*(*D_p_*) corresponding to the voltage distribution in Fig. 11 is plotted in Fig. 13. It is seen that a Gaussian distribution with a peak of 60.5 nm and a width of 4.9 nm has the same width as the measured distribution for *G*(*D_p_*)/*G*(*D_p_*)*_peak_* > 0.5, but the measured distribution is noticeably asymmetric towards the small particle sizes. Also one set of results is included in Fig. 13 where an attempt has been made to subtract the contribution of the doubly charged monomer concentration using the estimated charging probability. The corrected monomer distribution does not have a plateau.

## 7. Experimental Design and Statistical Analysis for Homogeneity, Best Estimate of Peak Diameter, and Repeatability Uncertainty

The experimental design for testing the homogeneity of the 60 nm and 100 nm samples, for determining the best estimate of the peak diameters, and for determining the uncertainty associated with repeatability is presented. The repeatability uncertainty, which is a so-called Type A uncertainty, is needed for computing the overall sizing uncertainty by combining with the Type B uncertainty discussed in the next section. The Type A uncertainties are those computed by statistical methods while the Type B uncertainties are computed by other means and are generally based on scientific judgment using all the relevant information available [4]. The relative uncertainty, which is the uncertainty divided by the mean value, will be used throughout this paper. The statistical analysis model is described in his section and the results of the analysis of variance (ANOVA) are presented.

### 7.1 Homogeneity Test—100 nm Spheres

The purpose of the homogeneity test was to verify at the 95 % confidence level that each bottle has the same peak size. The following experimental design was used. Nine bottles were selected at random from a lot of 150 bottles. On each of three different days, three bottles were assigned and two repeat measurements of peak voltage were performed from each bottle, for a total of eighteen measurements. The measurement plan is shown in Table 3. This sampling design is called a nested design [22], with bottles nested within a day, as opposed to a full factorial design, which would require that all nine bottles be measured each day. Taking this many measurements together with the control measurements was not feasible.

The procedure for determining the peak voltage was discussed in Sec. 6. The voltage peak is closely related to the peak in the particle diameter distribution. From the uncertainty in the peak voltage, the uncertainty in the particle diameter can be computed as discussed in the last part of this section.

To compensate for possible instrument drift within a day, the peak voltage of a control sample is measured, at the beginning of the series of measurements, at the midpoint, and at the end of the measurements. The peak voltage for the first two measurements, 92B and 42C as shown in Table 4, are divided by the peak voltage for the A^th^ run of the control sample, which was labeled as the 50^th^ sample. The next two sample measurements are divided by the middle control and the last two measurements by the last control. The same methodology is followed on the other two days. Causes of drift include changes in the ambient temperature and pressure.

To test for homogeneity, a two-factor analysis of variance was run with the factors being nested and random. The two random factors are: the day to day effects which are indexed by *i* and the bottle to bottle effects indexed by *j*. A random day effect means that if the measurements were performed on any day, observed daily fluctuations would look like a normal distribution. Replicate measurements are identified by the index *k*. Each of the 18 measured values of the normalized voltage are, therefore, denoted by *Vr_ijk_, i* = 1,2,3, *j* = 1,2,3, *k* = 1,2.

In a nested design analysis of variance model, the normalized voltages are assumed to be given by:

$$Vr_{ijk}=\mu_{..}+\alpha_{i}+\beta_{j(i)}+\varepsilon_{k(ij)},$$ (22)

where:

µ is a constant representing the component of peak voltage common to all measurements, which can also be thought of as the true value of the peak voltage, and is estimated by $\bar{V}r_{\ldots }$, the average of the 18 measurements of *Vr_ijk_*, where each dot represents an average over an index.
α*_i_* are *N*(0, *σ*_α_^2^), which is an abbreviation for normally distributed random variables with mean zero and variance *σ*_α_^2^. The quantity α*_i_* measures the random differences due to day to day variation.
*β_j_*_(_*_i_*_)_ are *N*(0,*σ*_β_^2^) random variables measuring variations from to bottle to bottle. The parentheses around the index *i* signify that the *j* index corresponds to a fixed day (nested within a day).
*ε_k_*_(_*_ij_*_)_ are *N*(0,*σ*_ε_^2^) random variables incorporating all other variation. The parentheses around *ij* signify that the *k* index corresponds to a fixed day and a fixed bottle.

Analysis of variance is based on the fact that the total sum of squares (SS) can be partitioned as follows:

$$\begin{array}{l} \sum_{i}\sum_{j}\sum_{k}{\left(Vr_{ijk}-\bar{V}r_{\ldots }\right)}^{2}=3\cdot 2\sum_{i}{\left(\bar{V}r_{i..}-\bar{V}r_{\ldots }\right)}^{2}\\ \;+2\sum_{i}\sum_{i}{\left(\bar{V}r_{ij.}-\bar{V}r_{i..}\right)}^{2}+\sum_{i}\sum_{j}\sum_{k}{\left(Vr_{ijk}-\bar{V}r_{ij.}\right)}^{2}, \end{array}$$ (23)

*SSTotal* = *SSDay* + *SSBottle*(*Day*) +*SSError*,

where,

$$\begin{array}{c} Vr_{\ldots }=\sum_{i}\sum_{j}\sum_{k}Vr_{ijk},\to \bar{V}r_{\ldots }=Vr_{\ldots }/18,\\ \bar{V}r_{ij.}=\frac{1}{2}\sum_{k}Vr_{ijk},\text{and}\bar{V}r_{i\ldots }=\frac{1}{6}\sum_{i}\sum_{k}Vr_{ijk}. \end{array}$$ (24)

Each of the individual sums of squares has associated degrees of freedom and expectations given in Table 4.

From the Law of Large Numbers, the mean squares (MS) are unbiased estimates of the expected value of the mean squares, E[MS], and thus provide criteria to test for either a day to day or a bottle to bottle effect (See Tables 6 and 8). To test the hypothesis that the data do not show a statistically significant bottle to bottle effect at the 95 % confidence level, one uses the F-ratio, F = MSBottle(Day)/MSError, and the hypothesis is not rejected if F ≤ F(0.95,6,9), the 0.95 quantile of the F distribution with 6 and 9 degrees of freedom. The computed value of F is 1.11 compared to a value of 3.37 for F(0.95,6,9). The hypothesis is not rejected, therefore, we treat the bottles as homogeneous.

To test the hypothesis that the data do not show a statistically significant day to day effect at the 95 % confidence level one uses the F-ratio, F = MSDay/MSBottle(Day), and we do not reject the hypothesis of no day effect if F<F(0.95,2,6). If there is no day to day effect, then $\sigma_{\alpha }^{2}=0$ and E[MSDay]/E[MSBottle(Day)] = 1. We find that F = 1.35 compared to a value of 5.14 for F(0.95,2,6). The hypothesis is not rejected. The data are treated as showing no day to day affect at the 95 % confidence level.

It is also important for the uncertainty analysis to compute the total experimental variance for this set of measurements. Since the F tests verify that there is no day to day effect or bottle to bottle effect, the standard formula for the variance of the mean for the 18 independent measurements is used

$$Var[\bar{V}r_{\ldots }]=\frac{1}{18}\frac{1}{17}\sum_{i=1}^{3}\sum_{j=1}^{3}\sum_{k=1}^{2}{\left(Vr_{ijk}-\bar{V}r_{\ldots }\right)}^{2}.$$ (25)

The resulting value of the variance of the mean is 2.16 ×10^−7^ with 17 degrees of freedom. The corresponding relative standard uncertainty in the mean, $u_{r}^{A}(\bar{V}r)$, is equal to 0.0465 %. The quantity $u_{r}^{A}(\bar{V}r)$ is equal to square root of the variance of the mean divided by the mean value, and this quantity will be reported in or the homogeneity and repeatability studies.

The last step is to relate the standard uncertainty for the voltage to the standard uncertainty in terms of particle diameter. From Eq. (2) and (6), one can obtain a relation between d*D_p_* and d*Vr*.

$$\frac{dD_{p}}{D_{p}}=\frac{dVr}{Vr}{\left(1-\frac{C^{\prime }(D_{p})D_{p}}{C(D_{p})}\right)}^{-1}.$$ (26)

Replacing the reduced differentials with the corresponding standard relative uncertainties, one obtains

$$u_{r}^{A}({\bar{D}}_{p})=u_{r}^{A}(\bar{V}r){\left(1-\frac{C^{\prime }(D_{p})D_{p}}{C(D_{p})}\right)}^{-1}.$$ (27)

Assuming a nominal temperature and pressure that is characteristic of the measurement condition, 24 °C and 100.7 kPa, the computed value of is 0.025 %.

### 7.2 Homogeneity Test—60 nm Spheres

As shown in Table 5, a nested experimental design was used to test the homogeneity of 150 bottles of 60 nm spheres. This design is similar to the design for the 100 nm spheres, except only two bottles were measured on each day. That is, 6 bottles rather than 9 were selected at random. A different pair of these bottles was measured each day for 3 consecutive days. Two repeat measurements from each bottle were done each day. The formulas and analyses are very similar to that above, but the indices and degrees of freedom for the sum of squares will change, as indicated in Table 6. A smaller number of samples were measured because of the increased complexity in generating smaller particles using electrospray generation rather than a pneumatic nebulizer, and because more control measurements were used. As indicated in Table 5, for the 60 nm particles, the same number of control measurements as sample measurements were used. In this way, the peak voltage measured for every bottle was divided by a unique control measurement; that is, the first control, 38A, is used to reduce the first sample, 39B, the second control, 38D, to reduce the second sample, 39C, etc. For the 100 nm spheres, each control measurement was used for two samples. Using a unique control measurement for each sample removed the co-dependency of the ratio resulting from using the same control on more than one sample.

The computed F value for the homogeneity test is F = 1.48 compared to a value of 4.76 for F(0.95,3,6). The hypothesis of no significant bottle effect is not rejected at the 95 % confidence level. Thus, the lot of 150 bottles can be treated as homogeneous.

The computed F value for the test of a day effect is F = 0.63 and compared to a value of 9.55 for F(0.95,2,3). The hypothesis of no significant day effect is also not rejected at the 95 % confidence level.

Since there is no day effect, nor a bottle effect, the variance of the average of the 12 independent *Vr* measurements is a good estimate with a resulting value of 7.58 ×10^−6^. The corresponding relative standard uncertainty in the mean, $u_{r}^{A}(\bar{V}r)$, is equal to 0.2753 %. Assuming a nominal temperature and pressure characteristic of the measurement condition, 21 °C and 99.8 kPa, the computed value of $u_{r}^{A}({\bar{D}}_{p})$ is 0.158 %.

The value of $u_{r}^{A}({\bar{D}}_{p})$ is about 6 times larger for the 60 nm spheres compared to the 100 nm spheres. Factors contributing to the larger variance for the 60 nm particles include the broader size distribution; the use of a less stable generator, electrospray rather than the pneumatic system; and the larger voltage uncertainty for the smaller particles.

### 7.3 Certification Measurements for 100 nm Spheres

The analysis of variance shows that there is no significant variation in the normalized peak voltage and thus in the peak particle size for the bottles tested. Therefore, calibrated measurements on a single bottle are considered sufficient to certify a value for the entire batch of 100 nm spheres. The certification measurements consisted of three repeat measurements on three separate days, all made for the particles from the same vial. In addition to the measurements of the unknown sample, a sample of the current SRM® 1963 was measured before and after each unknown, for the calibration of the DMA. The calibration process is explained in Sec. 6.1 and the measurement results are given in Table 7. Each calibration measurement was associated with only a single measurement of the 100 nm spheres to avoid co-dependency of the results.

The notation for the particle diameter measurements is *D_p_*(*ij*), where the index *i* (*i* = 1, 2, 3) is over days and the index *j* (*j* = 1, 2, 3) is over repetitions within a day. The one factor statistical model with random effects is given by:

$$D_{p}(ij)=\mu +\alpha_{i}+\varepsilon_{ij},$$ (28)

where:

µ.. is a constant representing the component of the peak diameter common to all measurements and is estimated by $\bar{D}p(..)$, the average of all twelve measurements of *D_p_*(*ij*) where each dot represents an average over an index.
*α_i_* are $N(0,\sigma_{\alpha }^{2})$ measuring differences due to day to day variation.
*ε_ij_* are $N(0,\sigma_{\varepsilon }^{2})$ random variables incorporating measurement repeatability and all other variation.

The ANOVA model is given in Table 8. The computed F value for the test of a day effect is F = 1.28 compared to a value of 5.14 for F(0.95,2,6). The hypothesis of no significant day effect is not rejected at the 95% confidence level.

Since there is no day effect, the variance can be estimated as the variance of the mean of the nine independent measurements of the peak diameter

$$Var({\bar{D}}_{p}(..))=\left(\frac{1}{9}\right)\left(\frac{1}{8}\right)\sum_{i=1}^{3}\sum_{j=1}^{3}{\left(D_{p}(ij)-D_{p}(..)\right)}^{2}.$$ (29)

The resulting value of the variance is 9.309×10^−4^ with 8 degrees of freedom and the value of $u_{r}^{A}({\bar{D}}_{p})=0.0300\%$.

### 7.4 Certification Measurements for 60 nm Spheres

The analysis of variance shows that there is no significant variation in the normalized peak voltage for the 60 nm spheres and thus in the peak particle size due to the bottles tested. Therefore, calibrated measurements on a single vial are considered sufficient to certify a value for the entire batch of the 60 nm spheres.

The experimental design for the 60 nm sphere measurement was similar to that of the 100 nm spheres. The measurement results for the diameter at the peak of the number distribution are given in Table 9 and the ANOVA model for this experimental design is given in Table 10. The major difference from the 100 nm measurements is that four measurements were made each day instead of three. The other change is a slight rearrangement of the measurement sequence for the calibration spheres, allowing an additional repeat measurement based on the same number of calibration measurements. For the 100 nm spheres, the first and last calibrations of the day were used with the first and last samples measured. The average of the second and third calibration was used for the middle measurement. For the 60 nm spheres, the first measurement used the first calibration, the second measurement used the second calibration, etc. In neither case was a calibration measurement used in normalizing more than one measurement to avoid codependency.

The computed F value for the test of a day effect is F = 2.57 compared to a value of 4.46 for F(0.95,2,8). The hypothesis of no significant day effect is accepted at the 95 % confidence level. Since there is no day effect, the variance can be estimated as the variance of the mean of the twelve independent measurements of the peak diameter. The resulting value of the variance is 13.20 ×10^−4^ with 11 degrees of freedom and the value of $u_{r}^{A}({\bar{D}}_{p})=0.0659\%$.

The Type A statistical results are summarized in Table 11 for the four sets of experiments. It is seen that for both particle sizes the homogeneity experiments and the certification measurements give relative standard uncertainties for the peak diameter within a factor of three. Also, in both cases, the uncertainties were at least a factor of two smaller for the 100 nm spheres compared to the 60 nm spheres. As discussed above, the major contributors to the larger uncertainty for the 60 nm spheres are thought to be the broader size distribution and the less steady operation of the electrospray generator compared to the pneumatic aerosol generator.

## 8. Type B Uncertainties/Propagation and Expanded Uncertainty

This section focuses on the uncertainties based on scientific judgment and on results from other studies. First, the key uncertainties are identified and then their effect on the uncertainty in particle size is obtained via propagation of uncertainty. Finally, the Type B uncertainty computed in this section is combined with the Type A in the previous section and a coverage factor estimated to give the expanded uncertainty, which corresponds to the 95 % confidence interval.

### 8.1 Uncertainty in Key Quantities

The values of the quantities needed for computing the peak particle diameter together with their uncertainties are summarized in Table 12. The methodology for estimating these uncertainties is given below.

#### 8.1.1 Pressure

The barometric pressure is measured using a Mensor Corporation Model 4011 digital pressure transducer containing an ion implanted silicon strain gage. The pressure transducer has a NIST traceable calibration with a relative combined standard uncertainty of ± 0.010 % over the range of 70 kPa to 140 kPa and a resolution of 0.0013 kPa (see Fig. 2). The pressure is measured in the monodisperse aerosol tube before the valve. The pressure drop across the monodisperse aerosol outlet slit at a monodisperse aerosol flow of 0.5 L/min was 3 Pa, which is small compared to the measurement uncertainty and is not corrected for.

#### 8.1.2 Temperature

Ultra stable thermistors provide accurate temperature measurements at two locations in the sheath flow. One thermistor is located in the upper sheath flow just before it enters the DMA and the other is located after the DMA exit. The thermistors are type CSP Thermoprobes manufactured by Thermometrics Incorporated, with NIST traceable calibrations with a combined standard uncertainty of 0.01 K There is an approximate 0.25 K temperature difference between the temperature of the sheath air entering the DMA and the temperature near the exit. As described in Sec. 4.3, the recirculating air is cooled to dissipate the heating from the pump and then flows through a copper coil to re-equilibrate to the ambient temperature. The slight temperature difference is a result of the equilibration not being complete. We estimate the temperature *T* within the analysis region as the average of the inlet and outlet temperatures *T_i_* and *T*_0_ and the standard uncertainty in *T* as $(T_{i}-T_{0})/2\sqrt{3}=0.072K$, assuming that the probability of the temperature is uniformly distributed over the interval *T_i_* to *T*_0_. The estimated uncertainty of the average temperature measurement for each voltage channel, 0.010 K, and the drift in temperature during a voltage scan, 0.010 K, are small compared to the temperature difference uncertainty and are neglected. From above, the nominal relative standard uncertainty of *T* based on an absolute temperature of 296 K is 0.025 %.

#### 8.1.3 Diameter for SRM® 1963

The certified number mean diameter for SRM® 1963 is 100.7 nm with an expanded uncertainty of 1.0 nm. The combined relative standard uncertainty, which is needed for the uncertainty analysis for the 60 nm and 100 nm spheres, is 0.47 % with 85 degrees of freedom [2].

#### 8.1.4 Voltage

The uncertainty in the peak voltage has three components: one arising from the discrete digital readout from the meter, another arising from its calibration, and the third arising from the ability to locate the peak.

The discretization uncertainty for the voltmeter is 0.5 *V*. The corresponding value of the relative uncertainty is estimated as 0.01 % for the 100 nm measurement case and 0.04 % for the 60 nm case, because the peaks are located at different magnitudes for the voltage, i.e. 3400 V for the 100 nm particles and 1400 V for the 60 nm particles.

As described in Sec. 4.4, the standard relative uncertainty in voltage due to calibration, which is carried out using an accurate 10 000 to 1 divider circuit (Spellman HUD 100 1 precision resistor ladder) and a high impedance digital voltmeter (Fluke Corporation 8060A digital multimeter), is estimated as 0.08 % over the range of voltages measured.

There are two factors in locating the peak. One is the choice of the fitting function and the other is the range over which the fit is made. The cubic function was used to allow a skewness in the shape of the distribution near the peak, which is not included in a Gaussian fit. Over the range of the fits, *N*/*N_p_* > 0.75, the Gaussian fit is essentially the same as a quadratic fit for finding the peak voltage. To assess the sensitivity to the fitting function, selected data sets (five data sets for each particle size) were also fit with a quadratic function and a quartic function. The data were fit over the ranges of about *N*/*N_p_* > 0.75 and *N*/*N_p_* > 0.85 to assess the sensitivity of the results to the fitting range, which also changed the number of points in the fitting. For the 100.7 nm SRM and for the new 100 nm standard, the average deviation of the quadratic and quartic fits relative to the cubic were both about 1.8 V and the range effect was also about 1.8 V. We estimated the combined standard uncertainty of these two components by the root-sum-of-squares and obtained 2.5 V, which equals a relative uncertainty of 0.07 % of 3400 V. For the 60 nm spheres, the average deviation of the quadratic and quartic fits relative to the cubic were about 13 V and 1 V and the range effect was 4.4 V for the quadratic compared to about 1.8 V for the cubic. Both of the large deviations observed for the quadratic fitting function are a result of the skewness in the distribution, which is not accounted for in the quadratic fit. Thus, the anomalous results for the quadratic fit are not relevant. Based on the range effect for the cubic (1.8 V) and the agreement between the cubic and quartic (1 V), we adopt the same estimate for the peak uncertainty, 2.5 V, as for the 100 nm spheres. Because of the lower nominal voltage for the 60 nm spheres (1400 relative to 3400), the relative uncertainty is increased to 0.18 %. The total voltage uncertainty is computed as the root-sum-of-squares of the three components with a result of 0.10 % for the 100 nm spheres and 0.20 % for the 60 nm spheres.

### 8.2 Propagation of Type B Uncertainties

The goal of the Type B uncertainty analysis for the 60 nm diameter and 100 nm diameter particles is to express the relative uncertainty of the diameter in terms of the relative uncertainties in the measured voltages, temperature, and pressure, the diameter of SRM® 1963; and the slip correction parameter *A*. The starting point is obtained from Eqs. (2) and (16),

$$D_{p}=\frac{C}{C_{s}}\frac{V}{V_{s}}D_{p}^{s},$$ (30)

where $D_{p}^{s}$ is the certified diameter of SRM® 1963 (100.7 nm). The differential of the particle diameter divided by itself can be expressed as:

$$\frac{dD_{p}}{D_{p}}=\frac{dC}{C}-\frac{dC_{s}}{C_{s}}+\frac{dD_{p}^{s}}{D_{p}^{s}}+\frac{dV}{V}-\frac{dV_{s}}{V_{s}}.$$ (31)

We note that neither the viscosity nor the electron charge differentials appear in this equation as they cancel out in the calibration measurement. If the calibration step was not conducted, then uncertainties in both of these quantities would also appear.

The slip correction factor *C* can be expressed as a function of the mean free path, particle diameter, and the slip correction parameter *A* as follows:

$$C=1+\frac{2\lambda }{D_{p}}A.$$ (32)

As above, the differential of *C* divided by *C* is:

$$\frac{dC}{C}=\left(\frac{C-1}{C}\right)\left[\frac{d\lambda }{\lambda }+\frac{dA}{A}-\frac{dD_{p}}{D_{p}}\right].$$ (33)

We note that the quantity *A* also has a small dependence on *λ* and *D_p_* that is not included here, see [11] for the extent of these quantities’ contribution. Substituting Eq. (33) into Eq. (31), we obtain:

$$\begin{array}{c} \frac{dD_{p}}{D_{p}}=\frac{dD_{p}^{s}}{D_{p}^{s}}\left(\frac{2C_{s}-1}{C_{s}}\right)+\frac{dV}{V}-\frac{dV_{s}}{V_{s}}+\frac{d\lambda }{\lambda }\left(\frac{C-1}{C}-\frac{C_{s}-1}{C_{s}}\right)\\ +\frac{C-1}{C}\frac{dA}{A}-\frac{C_{s}-1}{C_{s}}\frac{dA_{s}}{A_{s}}-\frac{C-1}{C}\frac{dD_{p}}{D_{p}}. \end{array}$$ (34)

This equation is then solved for *dD_p_*/*D_p_*,

$$\begin{array}{l} \frac{dD_{p}}{D_{p}}=\frac{f_{1}(C_{s})}{f_{1}(C)}\frac{dD_{p}^{s}}{D_{p}^{s}}+\frac{1}{f_{1}(C)}\left(\frac{dV}{V}-\frac{dV_{s}}{V_{s}}\right)\\ \;+\frac{f_{2}(C)-f_{2}(C)}{f_{1}(C)}\frac{d\lambda }{\lambda }+\frac{f_{2}(C)}{f_{1}(C)}\frac{dA}{A}-\frac{f_{2}(C_{s})}{f_{1}(C)}\frac{dA_{s}}{A_{s}}, \end{array}$$ (35)

where $f_{1}(C)=\frac{2C-1}{C}$ and $f_{2}(C)=\frac{C-1}{C}$.

The quantity *λ* is a function of pressure and temperature as indicated by Eq. (20). Recognizing that *λ*_0_ is a fixed quantity without uncertainty, the differential of the mean free path can be expressed as:

$$\frac{d\lambda }{\lambda }=\left(\frac{2}{T}-\frac{1}{T+S}\right)dT-\frac{dP}{P}.$$ (36)

The quantity *S* is the constant 110.4 K in Eq. (20). Substituting Eq. (36) into Eq. (35), we obtain the final result in terms of the measured quantities and in terms of the uncertainties in the diameter of SRM® 1963 and the uncertainty in the slip correction parameter,

$$\begin{array}{r} \frac{dD_{p}}{D_{p}}=\frac{f_{1}(C_{s})}{f_{1}(C)}\frac{dD_{p}^{s}}{D_{p}^{s}}+\frac{1}{f_{1}(C)}\left(\frac{dV}{V}\right)-\frac{1}{f_{1}(C)}\left(\frac{dV_{s}}{V_{s}}\right)\\ +\frac{f_{2}(C)}{f_{1}(C)}\frac{dA}{A}-\frac{f_{2}(C_{s})}{f_{1}(C)}\frac{dA_{s}}{A_{s}}+\frac{f_{2}(C)-f_{2}(C_{s})}{f_{1}(C)}\times \\ \left(2-\frac{T}{T+S}\right)\left(\frac{dT}{T}\right)-\frac{f_{2}(C)-f_{2}(C_{s})}{f_{1}(C)}\left(\frac{dP}{P}\right). \end{array}$$ (37)

Given that the variance of a sum of independent random variables is equal to the sum of the individual variances, and then treating all terms in Eq. (37) except the terms involving *A* as independent random variables, we have:

$$\begin{array}{l} u_{r}^{2}(D_{p})={\left(\frac{f_{1}(C_{s})}{f_{1}(C)}u_{r}\left(D_{p}^{s}\right)\right)}^{2}+{\left(\frac{1}{f_{1}(C)}u_{r}(V)\right)}^{2}\\ \;+{\left(\frac{1}{f_{1}(C)}u_{r}(V_{s})\right)}^{2}+u_{r}^{2}(D_{p};A,A_{s})\\ \;+{\left(\frac{f_{2}(C)-f_{2}(C_{s})}{f_{1}(C)}\left(2-\frac{T}{T+S}\right)u_{r}(T)\right)}^{2}\\ \;+{\left(\frac{f_{2}(C)-f_{2}(C_{s})}{f_{1}(C)}u_{r}(P)\right)}^{2}. \end{array}$$ (38)

The terms *A* and *A_s_* are dependent (correlated) and, thus, the associated variance, *u_r_*^2^ (*D_p_*; *A*, *A_s_*), includes a correlation term in addition to the sum-of-squares of the individual terms:

$$\begin{array}{r} u_{r}^{2}(Dp;A,A_{s})={\left(\frac{f_{2}(C)}{f_{1}(C)}\right)}^{2}u_{r}{}^{2}(A)+{\left(\frac{f_{2}(C_{s})}{f_{1}(C)}\right)}^{2}u_{r}{}^{2}(A_{s})\\ -2\left(\frac{f_{2}(C)}{f_{1}(C)}\right)\left(\frac{f_{2}(C_{s})}{f_{1}(C)}\right)\left(u_{r}^{Corr}(A)\right)\left(u_{r}^{Corr}(A)\right). \end{array}$$ (39)

In this expression, *u_r_*(*A*) is the relative standard uncertainty of *A*, *u_r_*(*A_s_*) is the relative standard uncertainty of *A_s_*, *u_r_*^Corr^(*A*) is the correlated contribution to the relative uncertainty of *A*, and *u_r_*^Corr^(*A_s_*) is the correlated contribution to the relative uncertainty of *A_s_*. To compute the quantity requires estimates for the *total combined* relative uncertainty in the slip correction parameter *A* and also estimates for the *correlated* contributions to the uncertainty. We estimate the correlated contribution as the combined relative Type B uncertainty, considering this contribution to be potential bias that has been introduced to the measurements. The Type B component used is computed by Kim *et al*. [12] and is based on propagating the effect of the uncertainty in each variable when computing the total uncertainty. The uncertainty in the calibration diameter is shown to be the major contributor to the uncertainty in *A*. If the true diameter were some amount larger than the measured diameter, then the value of *A* would be underestimated for all values of *A*. Our use of a calibration particle introduces the correlation effect since the values of *A* for both the unknown particle size (60 nm or 100 nm) and the SRM® 1963 would be shifted by almost the same amount from an error in the calibration diameter. The other Type B components of the uncertainty are likewise correlated. Values for the Type A and Type B uncertainties in the slip correction parameter *A* based on Eq. (25) in Kim *et al*. [12] are provided in Table 13. The computed value of *u_r_*(*D_p_*; *A*, *A_s_*), based on Eq. (39) with the correlation effect is noted to be seven times smaller for both the 100 nm spheres and the 60 nm spheres when compared to results obtained when ignoring correlation.

Next, we compute the relative Type B uncertainty based on Eq. (38) making use of the results in Tables 12 and 13. Table 14 presents each of the six terms expressed as uncertainties, i.e., the square root of the individual terms in Eq. (38). The combined uncertainty obtained by propagating the individual uncertainties is found to be 0.494 % for the 100 nm spheres and 0.469 % for the 60 nm spheres. The uncertainty of the diameter for the SRM® 1963 dominates the overall uncertainty with the next largest term being the slip correction factor term. It is important to note that if the correlation effect had not been accounted for, the slip correction would have been the dominant uncertainty and the value of *u_r_*(*D_p_*) would have increased by more than a factor of 1.8 to 0.92 % and 0.90 % for the 100 nm and 60 nm spheres, respectively.

### 8.3 Additional Uncertainty Issues

A number of other potential sources of uncertainty were examined including the effects of slip correction, flow ratio, size distribution width for the 100 nm spheres, flow matching, drift in the number concentration measurement, multiply charged spheres, a residue layer, and asymmetry in the size distribution of SRM® 1963.

#### 8.3.1 Slip Correction

The DMA data were analyzed using both the Kim *et al*. [12] expression for the slip correction and the Allen and Raabe expression [11]. The slip correction factor is used twice. First the factor is used with the calibration particles to determine the corrected flow. Secondly it is used with the concentration vs. voltage distribution of the unknown particle size to determine the peak particle size. For the 100 nm diameter particle size, the choice of the slip correction changes the particle size by about 0.02 nm—essentially no effect. This is because the size of the calibration particle is so close to the 100 nm sphere. In the case of the 60 nm spheres, the mean particle size increases by 0.38 % from 60.45 nm to 60.68 nm. We have chosen to use the Kim *et al*. expression [12] for the slip correction because it includes a quantitative uncertainty treatment. Allen and Raabe’s analysis does not include a treatment of the effect of the Type B uncertainties. The result based on the Allen and Raabe expression is presented because this expression has been widely used.

#### 8.3.2 Flow Ratio

Our analysis assumes that the ratio of the aerosol flow to sheath flow is 0.025. As discussed in Sec. 3.6, there is an estimated uncertainty of the ratio equal to + 2 %/− 7 %. The data from test g of the 60 nm spheres on February 16, 2005, was reanalyzed for the flow ratio reduced by 7 % and it was found that the particle sizechanged by 0.02 nm, which corresponded to a change of 0.03 %. This is a negligible effect.

#### 8.3.3 Width and Asymmetry of SRM® 1963 Size Distribution

Our analysis assumed that the standard deviation of the size distribution for SRM® 1963 was 2.0 nm. As indicated in Fig. 5, the DMA data are more consistent with a Gaussian size distribution with a standard deviation of 1.8 nm compared to 2.0 nm. In this case the effect of changing the width of the size distribution on the corrected flow was assessed for run g on September 20, 2004. The percentage change in the flow rate was − 0.01 %, which would produce a negligible change in the diameter of the 100 nm spheres.

For the Gaussian distribution, the number mean diameter and the peak diameter are the same. Measurements of the SRM size distribution carried out with the electrospray indicated a slight skewness in the distribution towards smaller particle sizes. This will result in the peak particle size being skewed to a larger size.

To determine the effect of this asymmetry on the measured diameter, we modeled the SRM® 1963 size distribution as an asymmetric Gaussian with the following form:

$$\begin{array}{l} G(D_{p})=\frac{N}{\sqrt{2\pi \sigma }}\exp\left[-\frac{{\left(D_{p}-D_{\mathrm{mod}}\right)}^{2}}{2{\left(\sigma +\Delta \right)}^{2}}\right]\;\text{for}\;D_{p}<D_{\mathrm{mod}}\\ G(D_{p})=\frac{N}{\sqrt{2\pi \sigma }}\exp\left[-\frac{{\left(D_{p}-D_{\mathrm{mod}}\right)}^{2}}{2{\left(\sigma -\Delta \right)}^{2}}\right]\;\text{for}\;D_{p}>D_{\mathrm{mod}}. \end{array}$$ (40)

This functional form was previously used by Ehara *et al*. [3] in their development of a method for computing the moments of the particle size distribution using the DMA. The quantity *D_mod_* is the mode diameter, which is the same as the peak diameter for the number distribution. The four parameters of the asymmetric Gaussian were determined for ten sets of SRM 963® size distribution data using a non-linear least square fitting algorithm developed by Ehara [23]. The average value and standard deviation of *Δ* were found to be 0.06 nm ± 0.05 nm and the average value of the standard deviation was 1.74 nm. The value of *Δ* is near the resolution limit of the measurement as indicated by the large value of the standard deviation of *Δ*. The nonlinear fit was for the normalized size distribution data points greater than 0.4. The average value for *Δ* was 0.05 based on the normalized size distribution data points greater than 0.6, but in this case the standard deviation in *Δ* increased by a factor of two. Because of the sensitivity to the fitting range, we estimate the uncertainty in *Δ* as 0.05 nm, which is the standard deviation of the 10 values of *Δ* obtained from fits of the normalized size distribution data points greater than 0.4, rather than using the standard deviation of the mean value of *Δ*.

**Fig. 14:**
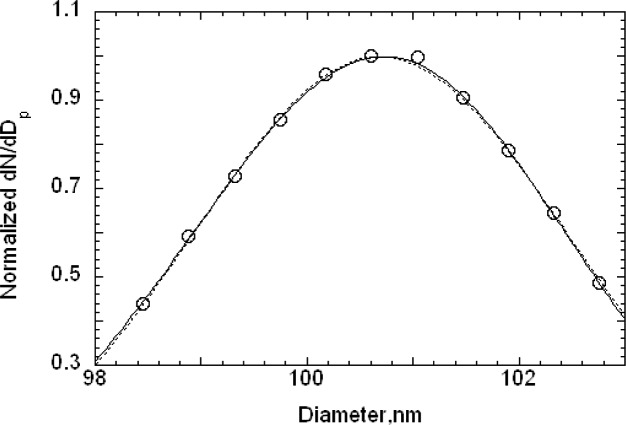
The size distribution is plotted for run H on February 18th with the asymmetric Gaussian the solid curve and the Gaussian the dotted curve. For the asymmetric Gaussian, *D_mod_* = 100.740 nm, *σ* = 1.730 nm, and *Δ* = 0.056 nm. For the Gaussian, *D_mod_* = 100.693 nm and *σ* = 1.733 nm.

Figure 14 shows that the asymmetric and symmetric Gaussian fits look almost identical for a data set with *Δ* = 0.056, which is close to the mean value of the 10 data sets. The asymmetric Gaussian peak diameter is 0.05 nm larger than the Gaussian, for smaller particle sizes the distribution is very slightly larger, and for the largest sizes the distribution is very slightly smaller than for the symmetric Gaussian. Fitting the data with a cubic equation, which also includes asymmetry near the peak, we find that the peak diameter is within 0.01 nm of the value for the asymmetric Gaussian. This figure provides a qualitative illustration of the resolution limit of the DMA, which detects an asymmetry in the distribution equal to only about 3 % of the standard deviation of the size distribution.

Equation (40) was used in place of the Gaussian distribution in Eq. (12) for the calibration of the DMA. The same method as described in Sec. 2.2 was used to carry out the analysis for two data sets for the 100 nm spheres and two sets for the 60 nm spheres. These analyses were carried out for *Δ* = 0.06, which is the mean value based on ten measurements, and for *Δ* = 0.11, which is the mean value plus the standard deviation. For a fixed number mean diameter equal to the certified value of 100.7 nm, the value of *D_mod_* for these two cases are 100.80 nm and 100.88 nm. Reanalyzing these data sets for the asymmetric Gaussian resulted in an increase in the measured size. For the 60 nm spheres, the average increase in diameter was 0.042 nm for *Δ* = 0.06 nm and 0.075 nm for *Δ* = 0.11 nm. For the 100 nm spheres, the average increase was 0.050 nm for *Δ* = 0.06 nm and 0.095 nm for *Δ* = 0.11 nm. The number of significant figures in the coefficients for the best fit equation was increased from 5 to 7 for one of the 100 nm data sets to obtain an accurate estimate of the change in the diameter. The results from the two data sets agreed within about 10 %. The values for *Δ* = 0.06 nm are used in adjusting the value of the final diameters in **Sec. 8.4**. The uncertainty in the correction, *u*(asym) is estimated as the difference between the changes in the particle diameters for *Δ* = 0.11 nm and *Δ* = 0.06 nm. The resulting values for *u*(asym) are 0.033 nm for the 60 nm spheres and 0.045 nm for the 100 nm spheres.

#### 8.3.4 Brownian Motion

In our analysis of the transfer function *Ω*, the effect of the Brownian motion is not included. As the particles move from the inlet to the outlet, the random Brownian motion will cause the particle trajectory to be perturbed. The resulting transfer function has been solved in closed form [24], and an explicit expressions for the peak value of the transfer function and its standard deviation have been obtained as a product of a diffusion dependent correction term multiplied by the expression without diffusion [25]. As seen in Table 15, the transfer function is broadened by a factor of 1.09 for the 100 nm spheres and by 1.21 for the 60 nm spheres.

The effect of diffusion on the peak particle size was assessed by first computing the concentration *vs* voltage distribution using Eq. (12) and then computing the distribution again using a version of Eq. (12) with the transfer function modified to include the effect of particle diffusion [23,24]. The size distribution *G*(*D_p_*) was then computed using Eq. (14) and the peak in the size distribution obtained. The parameters for the two Gaussian size distributions were number mean diameters of 101.60 nm and 60.70 nm and standard deviation of 2.6 nm and 4.3 nm, respectively. In both cases, the difference between the peak diameter with diffusion and without diffusion was negligible—less than 0.01 nm.

#### 8.3.5 Mismatch of Flow

The estimated mismatch in the sheath flow versus the excess flow is 0.01 % of the sheath flow. This slight mismatch means that the aerosol flow in, *Q_a_*, will be slightly different than the aerosol flow out, *Q_s_*. This will result in a transfer function with a trapezoidal shape rather than a triangular shape. The height of the trapezoid is equal to *Q_s_* / *Q_a_* in the case that *Q_s_* > *Q_a_*. For the above example, the peak in the transfer function is slightly reduced from 1.000 to 0.996. This is less of an effect than for Brownian motion where the peaks in the transfer function given in Table 15 are 0.78 (60 nm spheres) and 0.86 (100 nm spheres). Clearly, the flow mismatch effect is negligible.

#### 8.3.6 Drift in the Number Concentration

There are two sources of uncertainty in the number concentration measurement by the CPC (condensation particle counter). One is a random uncertainty and the second arises from a drift in the generator output. The random variability arises from the Poisson counting statistics for the aerosol flowing into the CPC. The uncertainty is equal to one divided by the square root of the number of particles counted over a 20 s averaging period for the measurement. For the lowest concentration measured, 125 part./cm^−3^, and a CPC flow rate of 5 cm^3^/s, the number of particles counted is 12,500, and the uncertainty is 0.89 %. The effect of this uncertainty is included in the Type A uncertainty determined from the repeat measurements of the peak particle size for the 60 nm spheres and the 100 nm spheres.

The drift in the number concentration of the 100 nm spheres using the pneumatic nebulizer is on average about 1 % and at most 2 % over the measurement interval of about 12 minutes. This drift has a negligible effect on the measured particle size. The drift in the number concentration of the 60 nm spheres and 100 nm spheres produced with the electrospray was larger with values up to 11 %. Measurements were made at a voltage close to the peak voltage at the start of the experiment, at the middle, and at the end to estimate a linear drift in the concentration over the course of the experiment for the experiments carried out with the electrospray. All the number concentration data were adjusted with the linear fit to give the estimated concentration at the time of the peak measurement. These adjustments were made for both the SRM calibration measurement used to find the corrected flow and for the 60 nm sphere measurements. The effect of the drift on the SRM calibration was a factor of about 3 smaller than for the 60 nm spheres because of the much narrower size distribution. The largest adjustment to the peak particle size was 0.189 nm and the average value of the absolute magnitude of the changes was 0.072 nm with a standard deviation of 0.063 nm. Seven adjustments resulted in an increase in the diameter, five a decrease, and the average diameter increased by 0.01 nm. We do not know the cause of the drift. It may be fortuitous that we observed about an equal number of plus and minus drifts. The linear drift correction is useful correction; however, more complex time variations were observed. While monitoring the concentration at a fixed voltage over a period of about 30 minutes, we observed a drift with two slopes in one case and a dip in concentration followed by a return to the original concentration in a second case. There is an uncertainty in the drift correction from such effects and we estimate this drift uncertainty as 0.036 nm, which is 1/2 the average of the absolute magnitudes of the changes. The corresponding relative uncertainty, *u_r_*(drift), is equal to 0.060 % for the measurement of the 60 nm spheres.

#### 8.3.7 Multiply Charged Spheres

In Sec. 6.1 it was pointed out that mobility of doublet trimers would overlap the mobility peak of the singlet monomers for the 100 nm spheres. A doublet trimer would appear at half the voltage of the same singlet trimer. The number concentration of singlet trimers in this overlapping region was found to be about one-tenth the concentration of the singlet monomers (run b, October 14, 2004, Fig. 8). The ratio of charging probabilities of a doublet trimer to a singlet monomer was estimated using Wiedensohler’s expression [16] and the surface area equivalent sphere for the trimer. Using this information, the number concentration of such doublet trimers for run b, September 20, 2004 (Fig. 4) was estimated and this number was subtracted for the total number concentration data for each voltage. The correction was about 1 % at the lower end of the size distribution and increased to almost 3 % at the upper end. The size distribution *G*(*D_p_*) was obtained from the net concentration data and the peak diameter was determined. The resulting diameter was 101.62 nm compared to the uncorrected value of 101.70 nm. This effect of 0.079 % is used as an estimate of *u_r_*(doublet), the Type B relative standard uncertainty resulting from the doublet trimer.

The effect of multiplet multimers is at least two orders of magnitude lower for the 60 nm spheres because of the much lower fraction of multimers produced, less than 1 %, when using the electrospray generator.

#### 8.3.8 Residue Layer from Impurities in PSL Suspension

The polystyrene spheres measured as an aerosol include a residue layer. This results from the evaporation of the droplet containing the polystyrene sphere. The nonvolatile contaminants from the dilution water and from the electrolyte and soluble fraction of the surfactant in polystyrene sphere suspension leave a residue layer over the sphere as the water evaporates. This will result in the sphere size as an aerosol being slightly larger than the diameter of the polystyrene sphere. Two methods for estimating the residue layer thickness are presented and the impact of this layer on the measured sphere size and on the uncertainty are presented.

##### 8.3.8.1 Direct Measurement of Residue

The measurement of the size distribution of the residue particles resulting from the evaporation of droplets not containing spheres provides a direct measurement of the residue thickness. It is assumed that the droplet distribution leading to the residue particles is the same as that for the droplets containing the spheres. The volume of the aerosol particles is the sum of the volume of the polystyrene sphere and the residue particle:

$$\frac{1}{6}\pi D_{a}^{3}=\frac{1}{6}\pi (D_{ps}^{3}+D_{r}^{3}),$$ (41)

where *D_a_* is the diameter of the aerosol particle, *D_ps_* is the diameter of the polystyrene sphere, and *D_r_* is the diameter of the residue particle. Solving for *D_ps_* in the limit (*D_r_* / *D_a_*)^1/3^ ≪ 1, one obtains:

$$D_{ps}=D_{a}\left(1-\frac{1}{3}{\left(\frac{D_{r}}{D_{a}}\right)}^{3}\right).$$ (42)

The size distributions of the residue particles are measured using the DMA. The conditions are identical to those used for the particle size distribution in terms of the solution concentrations and the operation conditions for the pneumatic generator. The conditions for the electrospray in terms of voltage were close but not identical to those used previously. The slight difference arose from optimizing the stability of the generator. In all cases the generator was operated in the pulsating mode with the current within 10 % of the value used during the certification measurements.

For the electrospray, there are less than 0.05 cm^−3^ residue particles counted by the condensation particle counter (CPC) for particle sizes less than 50 nm for the buffer and for SRM® 1963. The buffer solution contains ammonium acetate, which has a high vapor pressure and sublimes as an aerosol. This value is close to the minimum count rate for the condensation particle counter and about four orders of magnitude smaller than the peak particle concentrations for the SRM, (200 − 400) cm^−3^. The minimum particle size detected by the DMA/CPC combination for the flow settings used is about 12 nm. The residue particles are smaller than this size. An upper bound for the difference between *D_a_* and *D_ps_* is determined to be 0.06 nm from Eq. (42) assuming *D_r_* equal to 12 nm and *D_a_* = 100.7 nm.

**Fig. 15:**
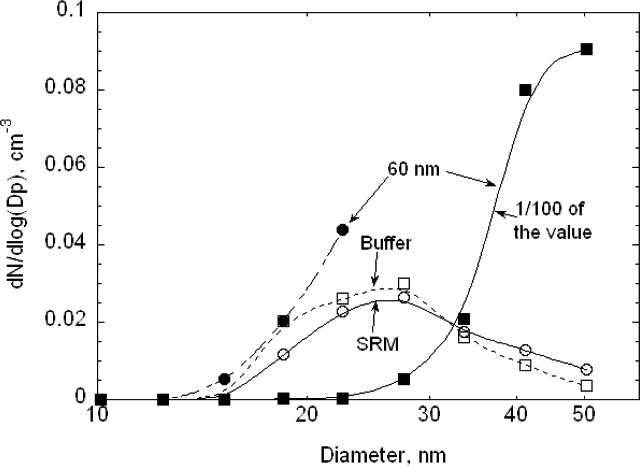
The logarithmic size distributions are plotted for the residues particles for the electrospray operated with the 100 nm SRM® 1963, the 60 nm spheres, and the buffer solution by itself. Two plots are included for the 60 nm spheres to show the rapid increase in the size distribution from the presence of the long tail in the size distribution of the 60 nm spheres.

The size distribution of the residue particles produced via the electrospray is similar for the 60 nm spheres, for SRM® 1963, and for the buffer solution for particles sizes less than 25 nm as indicated in Fig. 15. The logarithmic size distribution, d*N*/dln*D_r_*, is used because size distribution of sprayed liquids are typically better approximated by lognormal distribution than by a normal distribution. The upper bound of the residue size is again estimated as 12 nm. This results in an upper bound for the difference between *D_ps_* and *D_a_* equal to 0.16 nm assuming *D_a_* = 60.5 nm.

At a diameter of 27 nm, the size distribution for the 60 nm residue particles increases to a value of about 0.5 cm^−3^ (See Fig. 15) compared to a value of about 0.02 cm^−3^ for SRM® 1963 and the buffer. The distribution continues to increase rapidly with increasing particle diameter, while the distribution for the SRM® 1963 and buffer solution remain flat. The rapid increase for the 60 nm case is a result of the long tail in the 60 nm size distribution as indicated in Figs. 12 and 13.

**Fig. 16:**
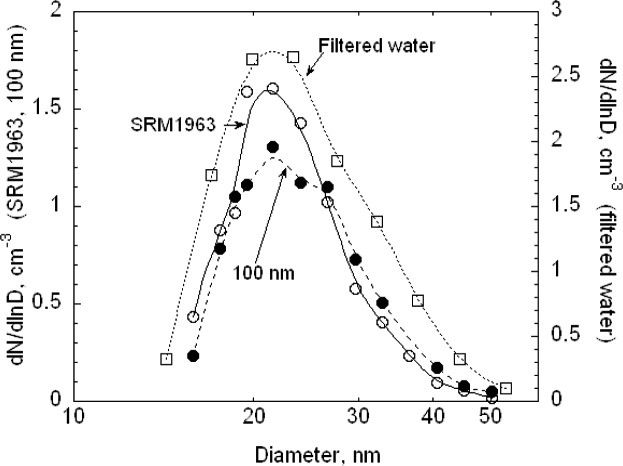
The logarithmic size distribution are plotted for the residue particles produced by the pneumatic nebulizer for the 100 nm sphere suspension and the SRM® 1963 suspension (left axis) and the filtered water (right axis).

**Fig. 17:**
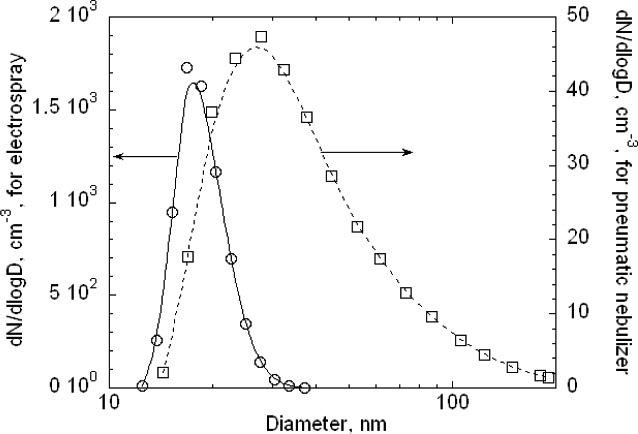
The number size distribution of residue sucrose aerosol from a 0.1 % mass fraction sucrose solution with an electrospray (left axis) and a pneumatic nebulizer (right axis).

For the pneumatic nebulizer, the apparent peak diameter is within a few percent of 21 nm for the dilution water, for the SRM® 1963, and for the 100 nm spheres as shown in Fig 16. The actual peak in the distribution is significantly smaller than 21 nm. The reason for the overestimate of the peak is that small residue particles generated by PSL suspensions are lost to the walls in the DMA or not counted by the CPC. In Fig. 17, the size distribution is plotted for the residue particles produced by nebulizing a 0.1 % mass fraction sucrose solution. In both the PSL sphere case and the sucrose cases, the number of droplets produced are expected to be similar and likewise the number of residue particles. However, the total number concentration of the residue particles produced is about 25 times smaller for the PSL suspension compared with the 0.1 % mass fraction sucrose suspension. It is inferred that most of the reduction is from the losses of small particles. If the residue particle size distribution for the PSL suspension is similar in shape to the distribution for the sucrose solution, the peak particle size would be at least a factor two smaller than the apparent size of 21 nm.

The probability of a droplet containing a PSL sphere is expected to be proportional to the product of the size distribution function d*N*/dln*D_p_* multiplied times the volume of the droplet which is equal to the volume distribution, d*V*/dln*D_p_*. The larger the droplet the more likely there is a polystyrene sphere in the droplet. This peak droplet size, *D_p_*(droplet), will produce a residue particle size, *D_p_*(residue), equal to the peak in the residue volume distribution. For the size distributions shown in Fig. 16, this size is about 25 nm. As discussed above, the actual peak in the residue size distribution is likely at least a factor of two smaller size resulting in a corresponding smaller value of *D_p_*(residue). A value of *D_p_*(residue) equal to 20 nm is an upper bound estimate. From Eq. (42) we find for the SRM® 1963 spheres and for the 100 nm spheres, that the difference between *D_a_* and *D_ps_* in both cases is 0.26 nm.

The previous analysis assumes that the probability of a PSL sphere being in a droplet is proportional to the volume of the droplet. This is a reasonable approximation if the droplet volume *V*(droplet) is much smaller than the average volume of the liquid per particle *V*(liq). If *V*(liq) is equal to *V*(droplet), this condition would break down with almost every droplet containing a particle. The residue particles would probably be dominated by the small size range of the droplet distribution. To estimate *V*(liq), the number concentration of spheres per volume of the liquid nebulized is calculated. The number concentrations *n_t_* of the 60.46 nm spheres and the 101.6 nm spheres in the vials with a nominal volume fraction of 0.005 are computed to be (4.3 × 10^13^ and 9.1 × 10^12^) cm^−3^ using the following equation:

$$n_{t}=\frac{6(0.005)}{\pi {\left(D_{p}\right)}^{3}}.$$ (43)

The suspensions are diluted by a factor of 38 for the 60 nm spheres (1 drop in 1.5 cm^3^ of water) and by 1000 for the 100 nm spheres (5 drops in 200 cm^3^ water) resulting in particle concentrations of (1.1 × 10^12^ and 9.1 × 10^9^) cm^−3^. The average volume of liquid per PSL sphere, computed as the reciprocal of the number concentration, is equal to 0.9 µm^3^ for the 60 nm spheres and 110 µm^3^ for the 100 nm spheres. At the end of Sec. 8.3.8.2, we compare this volume with the droplet volumes produced by the electrospray and the pneumatic nebulizer.

##### 8.3.8.2 Residue Estimate based on Droplet Size and Impurity Concentration

**Fig. 18:**
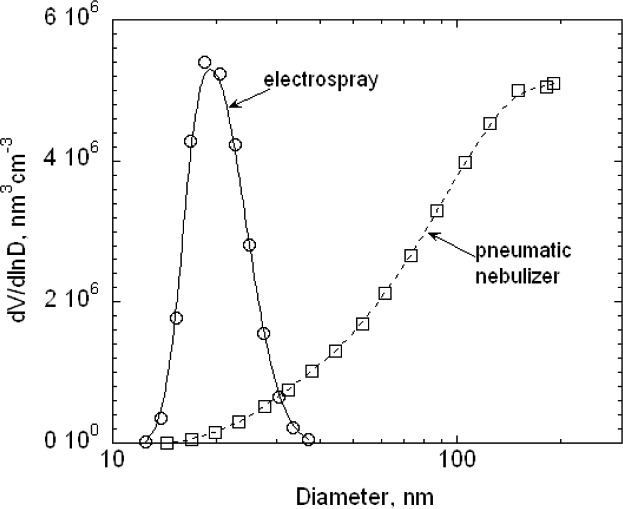
The volume size distribution of residue sucrose aerosol from a 0.1 % mass fraction sucrose solution with an electrospray and a pneumatic nebulizer.

A second method of estimating the residue effect is to determine the droplet size, and then from information on nonvolatile impurities, to estimate the residue particle size. It is seen in Fig. 17 that the number distribution of the aerosol produced by an electrospray with a 0.1 % mass fraction sucrose solution has a peak at about 17 nm, and a relatively narrow distribution with a geometric standard deviation of about 1.2. The number distribution produced by the pneumatic nebulizer has a peak size of about 27 nm and is much broader with a pronounced skewness toward the larger size particles (See Fig. 17). The peak in the volume distribution is about 19 nm for the electrospray and is about 200 nm for the pneumatic nebulizer as seen in Fig. 18.

The droplet diameter is often assumed to be related to the sucrose residue diameter by the relation [26,27]:

$$D_{p}(\text{droplet})={\left(c/\rho_{s}\right)}^{-1/3}D_{p}(\text{sucrose}\;\text{residue})$$ (44)

where *c* is the mass fraction of sucrose in the solution and *ρ*_s_ is the density of sucrose (1.58 g cm^3^). Recent work by Stanley Kaufman [28] indicated this to be the case for the electrospray, but that a 1/5th power dependence appeared more valid for pneumatic type nebulizers. Based on a 19 nm residue size and a sucrose mass fraction of 1.0 × 10^−3^, the estimated droplet size for the electrospray is 220 nm.

The estimate for the pneumatic nebulizer is more approximate, since the data does not reach the peak. It does appear to be close to the peak based on the flattening of the data and the peak is estimated as 200 nm. Based on a 1/5th power law, the predicted droplet size is 800 nm. If we assumed a 1/3rd power law, the resulting peak size would be 2300 nm.

The volume of the droplets corresponding to the volume mean diameter are 6.4 µm^3^ and 5.6 × 10^−3^ µm^3^. Dividing the volume of liquid per particle computed in the previous section by the droplet volume, one finds in both cases that the average volume of liquid per sphere is much greater than the droplet volume (160 times larger for the 60 nm sphere and 17 times larger for the 100 nm spheres). Thus the assumption that the probability of a particle being in a sphere is proportional to the droplet volume is reasonable.

To estimate the residue size from the droplet, we need an estimate of the non-volatile impurities in the PSL suspension. For the 100 nm spheres, the mass fraction of the non-volatile material included 2.1 × 10^−4^ surfactant and 6.0 × 10^−5^ electrolyte remaining after the synthesis. Assuming half of the surfactant on the particle surface, the total mass fraction of non-volatile material in the liquid is 1.7 × 10^−4^. The mass, *m*_i_, of the non-volatile impurities in the five droplets added to 200 ml of filtered deionized water is estimated based on an estimated droplet size of 0.040 cm^3^:

$$m_{i}=5(0.040)(1.7\times 10^{-4})=3.4\times 10^{-5}g.$$ (45)

The mass fraction of these nonvolatile impurities in the 200 ml of water is 1.7 × 10^−7^. To obtain the total mass fraction of non-volatile impurities one must add the approximately 1 × 10^−6^ mass fraction [27] resulting from the impurities in the filtered-deionized water. Assuming a density of the impurities deposited on the sphere after evaporation of the water of 1.5 g/cm^3^, the resulting volume fraction of non-volatile impurities, *V_r_* is 7.8 × 10^−7^.

The increase in the PSL sphere diameter Δ*D_p_* resulting from the impurity can be estimated based on the volume fraction of the impurities and the diameters of the droplet from the pneumatic atomizer (2000 nm) and the PSL sphere (101.6 nm) using the following formula[27]:

$$\Delta D_{p}=\frac{V_{r}D^{3}(droplet)}{3D_{ps}^{2}}=0.30\;\text{nm}.$$ (46)

This value of 0.30 nm is close to the estimate of 0.26 based on the residue particle size.

For the SRM® 1963, there was no surfactant added to the suspension. In this case the estimated value of *V*_r_ is about 7.1 × 10^−7^ and the estimated thickness is reduced slightly to a value of about 0.27 nm.

For the 60 nm spheres, there is no surfactant added and there is no information regarding other non-volatile impurities. We estimate the mass fraction of the impurities in the droplet to be 6.0 × 10^−5^, which is equal to the mass fraction of electrolyte impurities in the 101.6 nm spheres. In this case there is one drop of PSL spheres in 1.5 cm^3^ of filtered-deionized water. Including the impurities in the dilution water, 1 × 10^−6^, the mass fraction of the diluted solution is 2.6 × 10^−6^ and *V*_r_ is 1.7 × 10^−6^ assuming a density of 1.5 g/cm^3^. From the formula above, we find *ΔD_p_* = 0.002 nm. This is more than an order of magnitude smaller than the upper bound estimate of 0.12 nm based on the residue particle size. The residue diameter corresponding to a 220 nm droplet produced by the electrospray and a volume fraction of 1.7 × 10^−6^ would be 3.0 nm, which is well below the smallest size measurable by the DMA used in this study. Thus it is not surprising that the value of *ΔD_p_* estimated from the residue size is a gross over estimate. Realizing that there is a large uncertainty in the contaminant concentration, a more realistic upper bound estimate of *ΔD_p_* is 0.01 nm for the 60 nm spheres, which corresponds to a contaminant volume fraction *V*_r_ = 8.5 × 10^−6^. From Eq. (46), the value of *ΔD_p_* for the SRM® 1963 aerosol produced by the electrospray is 0.003 nm assuming the same contaminant concentration as the 60 nm spheres.

##### 8.3.8.3 The Impact of the Residue Layer on the Measured Peak Particle Sizes and Uncertainties

The estimated value of *ΔD_p_* from the residue for the 100.7 nm SRM® 1963 varies for the following three cases: the original measurement of SRM® 1963, the measurement using the pneumatic atomizer, and the measurement involving the electrospray. In the original measurements of SRM® 1963 [2] an impactor was used after the nebulizer to remove the larger droplets. The estimated residue increase to the diameter [27] was 0.15 nm and the uncertainty range from the residue effect on diameter is given as 0 % / 0.3 % of the mean diameter. An alternative statement of this result adopted here is that *ΔD_p_*(orig) = 0.15 nm ± 0.15 nm. For the current measurements with SRM® 1963 using the nebulizer, the estimated value of *ΔD_p_*(Neb) = 0.26 nm, and for the measurements with the electrospray, *ΔD_p_*(ES) = 0.003 nm. The uncertainties in these estimates are large. We assume that the uncertainty for the nebulizer case is the same as for the original study, ± 0.15 nm, and that for the electrospray it is equal to the value of *ΔD_p_*, ± 0.003 nm.

The three different values of *ΔD_p_* mean that the size of the SRM® 1963 as an aerosol is different in each of the three measurements. For the original measurement [2], the peak aerosol size, $D_{a}^{orig}(peak)$, for SRM® 1963 is 100.7 nm. For the other two cases, the peak aerosol size and the uncertainty associated with the residue measurement, *u*(residue), are given by:

$$D_{a}^{i}(peak)=D_{a}^{orig}(peak)+\Delta D_{p}^{i}-\Delta D_{p}^{orig}$$ (47)

$${\left(u^{i}(residue)\right)}^{2}={\left(\frac{\partial D_{a}^{i}(peak)}{\partial \Delta D_{p}^{i}}\right)}^{2}\Delta^{2}D_{p}^{i}+{\left(\frac{\partial D_{a}^{i}(peak)}{\partial \Delta D_{p}^{orig}}\right)}^{2}\Delta^{2}D_{p}^{orig},$$ (48)

where i = 1 corresponds to the pneumatic atomizer and i = 2 to the electrospray. The following values for the peak diameter and uncertainty resulting from the residue layer are given by: (100.81 ± 0.21) nm for the pneumatic nebulizer and (100.55 ± 0.15) nm for the electrospray.

The change in the size of the calibration particle will in turn cause a change in the diameter of the 100 nm spheres and the 60 nm spheres. To compute this change, the predicted number concentration of the aerosol exiting the DMA is computed as a function of voltage using Eq. (5) based on the revised size of 100.55 for the SRM® 1963 particles for the 60 nm spheres and 100.81 for the 100 nm spheres. The peak voltage is computed and the corrected flow determined as discussed in Sec. 2.2. The resulting change in the mobility is computed using Eq. (15) and then the change in diameter is computed from the mobility using Eq. (2). The computed change for the 60 nm spheres diameter is − 0.114 nm and for the 100 nm sphere diameter is 0.110 nm. The computed uncertainties in the diameters resulting from the residue layer uncertainty for the SRM are ± 0.114 nm for the 60 nm spheres and ± 0.206 nm for the 100 nm spheres.

### 8.4 Bias Correction, Total Type B Uncertainty, and Expanded Uncertainty

Slight corrections are made to the sizes of the 100 nm and 60 nm spheres as a result of bias effects from the asymmetry in the size distribution of the SRM® 1963 spheres, *β* (asym), and from the effect of the residue layer on the SRM® 1963 spheres, *β* (residue). The corrected diameter, *D_p_*(corr), is computed based on the following sum:

$$D_{p}(corr)=D_{p}(peak)+\beta (asym)+\beta (residue).$$ (49)

The resulting values for *D_p_* (corr) for the 100 nm spheres and the 60 nm spheres are 101.76 nm and 60.39 nm (Table 16).

The total relative type B uncertainty, $u_{r}^{\text{Total}\;\text{Type}\;B}$, for the 60 nm spheres is estimated as the root-sum-of-squares of *u_r_*(*D_p_*), *u_r_*(asym), *u_r_*(drift), and *u_r_* (residue), all given in Table 17. The resulting value of $u_{r}^{\text{Total}\;\text{Type}\;B}$ is 0.497 %. For the 100 nm spheres, $u_{r}^{\text{Type}\;B}$ is estimated as the root-of-squares of *u_r_*(*D_p_*), *u_r_*(asym), *u_r_*(doublet), and *u_r_*(residue). The resulting value of $u_{r}^{\text{Total}\;\text{Type}\;B}$ is 0.540 %.

The total Type A and Type B standard uncertainties are combined as a root-sum-of-squares to obtain the combined relative standard uncertainty of the particle diameter, *u_combined_* = 0.540 % for the 100 nm spheres and 0.512 % for the 60 nm spheres. We wish to compute the expanded uncertainty, *U*(*D_p_*), defined such that there is an approximately 95 % (95.45 %) level of confidence that the true diameter is within ±*U*(*D_p_*) of the measured diameter. For the case where there is a large number of degrees of freedom and a normal distribution applies, *U*(*D_p_*) is 2 *u*(*D_p_*), where the factor 2 is called the coverage factor. The Type A uncertainty for the measurement of particle diameter has 8 degrees of freedom for the 100 nm spheres and 11 degrees of freedom for the 60 nm spheres. The uncertainty in SRM® 1963, $u_{r}(D_{p}^{S})=0.47\%$, is a Type B uncertainty and has 85 degrees of freedom [2]. For all the other Type B uncertainties, we assign an infinite number of degrees of freedom. Because the estimate of the standard uncertainty for these components does not involve statistical analysis, any estimate of the number of degrees of freedom would be fanciful. A summary of the various uncertainty components and associated degrees of freedom are given in Table 18.

To compute the expanded uncertainty we first compute the effective degrees of freedom for the combined uncertainty and then determine the coverage factor *k_p_* based on the Student’s t-distribution. We use the Welch-Satterthwaite formula [29] to compute the effective degrees of freedom, *ν_eff_* :

$$\nu_{\;eff}=\frac{u^{4}(D)}{\sum_{i=1}^{N}\frac{c_{i}^{4}u_{i}^{4}(D)}{\nu_{i}}}=\frac{u_{combined}^{4}\left(D_{p}\right)}{\frac{u_{A}^{4}}{8}+\frac{u_{r}^{4}\left(D_{p}^{s}\right)}{85}}.$$ (50)

The expression on the right hand side of Eq. (50) provides the explicit expression for the case *D_p_* = 101.76 nm. The Type B uncertainties with assumed infinite number of degrees of freedom do not contribute to the sum. Also, the sensitivity factors, *c_i_*, are unity in our case where the uncertainty is expressed as a relative uncertainty. Substituting the appropriate values in Eq. (50), we obtain *ν_eff_* = 153 and 123 for the 100 nm spheres and the 60 nm spheres. The coverage factors based on the Student’s t-distribution are 2.0165 for 153 degrees of freedom and 2.0205 for 123 degrees of freedom for the 95.45 % confidence level. Even though the repeatability degrees of freedom are small, the final result for *k_p_* is close to the value of 2.0 for infinite degrees of freedom because the uncertainty is dominated by the Type B uncertainty, which is assumed to have infinite degrees of freedom for each component except for the value of SRM® 1963. The resulting expanded relative uncertainties are 1.10 % for the 100 nm spheres and 1.04 % for the 60 nm spheres.

If the correlation effects in the slip correction had not been accounted for, then the resulting increased value of $u_{r}^{2}(D_{p})$ (see last paragraph of Sec. 8.2) would have resulted in an expanded relative uncertainty of about 1.8 % for both the 100 nm spheres and the 60 nm spheres.

## 9. Test for Multimers

**Fig. 19:**
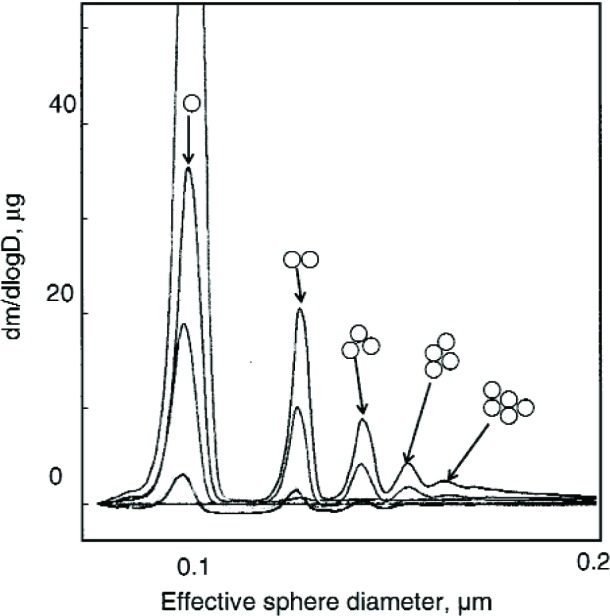
The mass distribution versus effective sphere diameter obtained by centrifugation showing agglomeration of SRM® 1963, evidenced by the relative amounts of monomer, dimer, trimer, tetramer, and pentamer. Results are presented for five samples for which the fraction agglomerated varied from less than 1 % for one sample to as much as 97 % for two of the samples.

Many of the previous SRM® 1963 samples had experienced a significant amount of agglomeration. Centrifuge measurements [1] showed that there were large populations of multimers in four of five samples analyzed (see Fig. 19). The ratio of the number concentration of dimers to monomers in these four samples was in the narrow range from 0.26 to 0.29. In one sample the ratio of multimers to monomers was 0.29 (dimer), 0.08 (trimer), 0.03 (tetramer), and 0.01 (pentamer). In these four samples, the fraction of the total mass of multimers ranged from 65 % to 97 %. Also, in these cases there were flocs large enough to be visible to the eye and they did not deaggregate during ultrasonic treatment.

Dynamic light scattering (DLS) measurements were also shown to detect the presence of multiplets in selected samples. The hydrodynamic diameter, which is measured by DLS, is a factor of 1.39 larger for a dimer compared to a monomer [30]. For six samples with large visible flocs, the apparent size obtained by dynamic light scattering ranged from 124 nm to 172 nm. The very large visible flocs were removed by a 1.2 µm pore diameter filter before making the measurements. For three samples without visible aggregates, the dynamic light scattering diameter was in the range of 107 nm to 110 nm. We believe these samples have a minimal amount of agglomerates. The large difference between the DLS sizes for the agglomerated sample compared to the unagglomerated sample is the basis for our test for agglomeration for the 100 nm and the 60 nm samples.

We have performed dynamic light scattering (DLS) measurements on samples of the 100 nm spheres and 60 nm spheres. From the DLS measurements, the particle diffusion coefficient, *D_f_*, is determined. This coefficient is, in turn, related to the particle’s hydrodynamic diameter, *D_h_*, via the Stokes-Einstein equation:

$$D_{f}=kT/(3\pi \eta D_{h}),$$ (51)

where *k* is the Boltzmann constant and *η* is the viscosity of the medium at the absolute temperature T, with a value of 1.00 × 10^−5^ kg/m^2^ for water at 20.0 °C. The hydrodynamic diameter size is typically slightly larger than the geometric diameter size as a result of adsorption of water on the particle surface and due to the effect of the particle’s electrical double-layer in the surrounding medium.

The principle of operation of DLS is that the random diffusive motion of particles of order 1 µm or smaller causes a time variation in the scattered signal on time scales of order 1 µs. For example, at one instant two particles may be positioned so that the scattered light from both is in phase and then at a later time the scattered light could be out of phase due to Brownian movement on the order of the wavelength of light. The smaller the particle diameter, the larger the diffusion coefficient and the faster the modulation of the scattered intensity.

To process this rapidly changing light scattering signal, a photon correlator is used where each channel counts photons arriving over a particular time span. The intensity autocorrelation function is obtained by multiplying the photon count at time *t* by the count at time *t* + *τ*, where *τ* is the delay time. For delay times over which the particles move only a small fraction of the wavelength of light (time on the order of 100 ns), the degree of correlation is high. At long times, when there is no correlation, the product of the two intensities approaches the square of the time-averaged scattering intensity. Generally, the autocorrelation function is normalized to this long-time product.

For a monodisperse distribution of independent scatterers, the normalized intensity autocorrelation function, *G*(*τ*), is an exponential function dependent on the diffusion coefficient *D_f_* and the wave vector magnitude *q*:

$$\begin{array}{l} \;G(\tau )=B+C_{1}\exp\left(-2\tau D_{f}q^{2}\right),\\ \text{with}\;q=\frac{4\pi \;n_{0}}{\lambda }\sin(\theta /2), \end{array}$$ (52)

and where *B* is the baseline (long-time value of *G*(*τ*)), *C*_1_ is an instrumental coherence factor, *λ* is the wavelength of light, *θ* is the scattering angle, and *n*_0_ the refractive index of the liquid. In our case the wavelength of light is 633 nm (HeNe laser), the refractive index of water is 1.33, and the scattering angle 90°.

For polydisperse size distributions, the autocorrelation function is expressed as an average over the distribution of particle masses where *f* (*m_p_*)*dm_p_* is equal to the number fraction of particles with mass between *m_p_* and *m_p_* + *dm_p_*.

$$G(\tau )=B+\sigma |\int f\left(m_{p}\right)m_{p}^{2}\exp\left(-2q^{2}D_{f}\left(m_{p}\right)\tau \right)dm_{p}|,$$ (53)

where the factor $m_{p}^{2}$ accounts for light scattering intensity being proportional to the square of the particle volume. A widely used method for analyzing the data is to expand ln *G*(*τ*) of the autocorrelation function in a Taylor series about *τ* = 0 resulting in the expression [31,32]:

$$\ln G(\tau )-B=\ln\sigma -2D_{fz}q^{2}\tau +\delta D_{fz}q^{4}\tau^{2}+\ldots ..$$ (54)

The quantities *D_fZ_*, the *z*-average [31] diffusion coefficient, and *δ*D*_fZ_*, the variance of the *z*-average diffusion coefficient, are defined below:

$$D_{fZ}=\frac{\int D_{f}\left(m_{p}\right)m_{p}^{2}f\left(m_{p}\right)dm_{p}}{\int m_{p}^{2}f\left(m_{p}\right)dm_{p}},$$ (55)

$$\delta D_{fZ}=\frac{\int {\left(D_{f}\left(m_{p}\right)-D_{fZ}\right)}^{2}m_{p}^{2}f\left(m_{p}\right)dm_{p}}{\int m_{p}^{2}f\left(m_{p}\right)dm_{p}}.$$ (56)

Since the diffusion coefficient is inversely proportional to the hydrodynamic diameter, *D_h_*, the moments can be expressed in terms of *D_h_*. Equation (55), for example, becomes

$$\frac{1}{{\langle D_{h}\rangle }_{Z}}=\frac{\int D_{h}^{5}f(D_{h})dD_{h}}{\int D_{h}^{6}f(D_{h})dD_{h}}.$$ (57)

Our measurements were carried out with a Malvern Zetasizer 3000HS. The samples consisted of one or two drops of the PSL samples diluted in 60 cm^3^ or 80 cm^3^ of filtered, deionized water. The samples were ultrasonicated for about 10 s and then filtered with a 1.2 µm pore size filter before transfer to the sample cell. Filtering removed large flocs and miscellaneous dust particles. The transfer was performed in a laminar-flow clean-air hood. Typically each measurement was carried out for 60 s, and six repeat measurements were made for each sample.

For the 60 nm spheres, three different samples were run each day, and a sample of the 100 nm SRM^®^ 1963 was run at the beginning and end of the measurement sequence. The 100 nm sample was selected based on screening 10 SRM® 1963 samples to find the one with the smallest peak size. The major findings were little sample-to-sample variability for 〈*D_h_*〉*_Ζ_* (see Table 19) for each sample (range from 59.3 nm to 63.4 nm) and an average of the six values of 〈*D_h_*〉*_Ζ_*, ${\langle {\bar{D}}_{h}\rangle }_{Z}$, equal to 60.5 nm ± 1.5 nm compared to 60.39 nm measured by the DMA. This indicates at most a couple percent of dimers, trimers, or larger multimers. The hydrodynamic diameter of a dimer by DLS is 84 nm, a factor of 1.39 times larger than a monomer [2]. Also, there is no indication of a much larger presence of dimers in one bottle compared to another. This data is important for future comparisons to assess whether agglomeration is taking place in the samples over a period of time, as was the case with SRM® 1963.

For the 100 nm spheres, three samples were measured, and for two of the samples, the measurements were made at two different concentrations (See Table 20). For this instrument, the optimum concentration results in a photon count rate in the range of (50,000 to 200,000)s^−1^. We observed little change in 〈*D_h_*〉*_Ζ_* over the range (40,000 to 600,000)s^−1^. The value of ${\langle {\bar{D}}_{h}\rangle }_{Z}$ is 99.8 nm compared to the DMA size of 101.6 nm. There is no evidence of formation of dimers or trimers based on this result.

The polydispersity index, which is the variance divided by the square of 〈*D_h_*〉_Z_, is about 0.070 for the 60 nm spheres and 0.033 for the 100 nm spheres. These polydispersity indices correspond to relative standard deviations of 0.26 and 0.18 compared to values of 0.076 and 0.026 for the 60 nm and 100 nm spheres sized by the DMA. It was recognized as early as 1972 [33] that it is difficult to make accurate measurement of the polydispersity index by dynamic light scattering. Weiner and Tscharnuter [34] demonstrated through a numerical example that a change in the baseline by 0.1 % and by 0.3 % increased the polydispersity from an assumed value of 0.0 to 0.02 and to 0.056. These values correspond to relative standard deviations of 0.14 and 0.24. So it is clear that accurate width measurements are not possible by dynamic light scattering. Still, the polydispersity index provides a qualitative indication that the distribution of the 100 nm spheres is narrower than the 60 nm spheres. For the most agglomerated samples of SRM® 1963, with *D_Z_* in the range (150 to 170) nm, the polydispersity index is in the range 0.15 to 0.17. This corresponds to a relative standard deviation of about 0.40 %. This value is clearly larger than the value for the 60 nm and 100 nm spheres, and can be used as supportive information regarding the presence of agglomerates.

Our results demonstrate that for both the 100 nm spheres and the 60 nm spheres, there is little if any agglomeration to form dimers, trimers, etc. Periodic DLS measurements on samples would be useful to assess whether dimers are starting to form. If 5 % of the 60 nm monomers became dimers, then, according to Eq. (57) and the 84 nm hydrodynamic diameter of a dimer, the measured hydrodynamic diameter of the suspension would increase by 3.7 %. The reported [35] sizing repeatability and accuracy for monodisperse polystyrene spheres are about ± 2 %. So detecting a small change in a measured, 〈*D_h_*〉*_Z_*, especially if the same instrument is used as in the original measurements, should be feasible.

## 10. Other Measurements of the 100 nm Spheres and the 60 nm Spheres

**Fig. 20:**
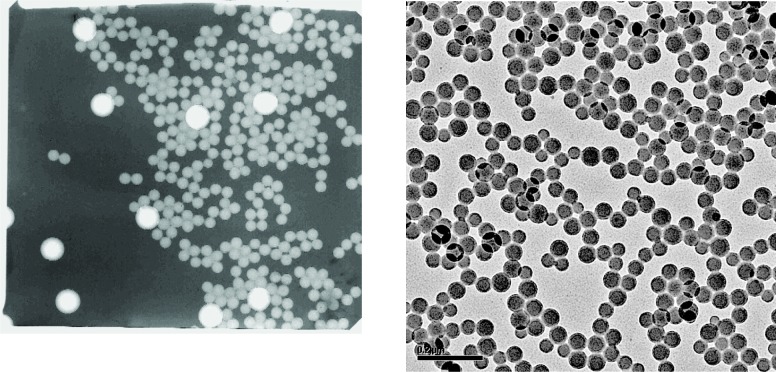
Transmission electron microscope images of the 100 nm spheres (left) and 60 nm spheres (right). The 205 nm spheres were deposited along with the 100 nm spheres by Duke Scientific Co. to provide a magnification calibration. The magnification for the TEM image of the 60 nm spheres taken by JSR Co. is indicated with a 200 nm length scale in the bottom left of the image.

The size distribution of the 100 nm spheres were measured by Duke Scientific [36] using three methods. The number mean size and standard deviation of the size distribution based on sizing 386 spheres by transmission electron microscopy were 98.9 nm and 2.8 nm. Images of the 100 nm spheres along with 205 nm calibration spheres are shown in Fig. 20. The 205 nm spheres were used to calibrate the magnification of the TEM. The number mean diameter obtained by use of the DMA was 100.7 nm and the z-averaged diameter obtained by dynamic light scattering was 101 nm based on six repeat runs. Results were not obtained on the peak diameter. In the next section the number mean diameter results will be computed for the 100 nm spheres and compared with the Duke Scientific measurements.

A TEM image of the 60 nm spheres obtained by JSR Company is shown in the lower portion Fig. 20. It is evident from the micrograph that the size distribution of the 60 nm spheres is broader than the 100 nm spheres.

## 11. Discussion

The discussion focuses on two topics. The first is concerned with the applications of these calibration standards. The second focuses on uncertainty issues that will arise for smaller calibration sizes.

### 11.1 Calibration Opportunities/Issues

The two calibration standards will be certified and then released by NIST as standard reference materials. We believe that these particles will be useful in a number of applications demanding an accurate sizing calibration standard. Here, we discuss four such applications. The first application is as a magnification calibration standard for transmission electron microscopes. The method has been used successfully in the past, but there is a possibility of electron beam damage to the PSL spheres especially for the 60 nm spheres with the smaller polymer lengths. There have been a number of studies of the effect of the exposure time on the shrinkage of the particle size. Methods of correcting for this effect have been developed based on the use of several exposure times [37]

A second application is the calibration of instruments for measuring particle size in liquids. One such measurement is dynamic light scattering, which was discussed in Sec. 10. The standard precaution of filtering the sample with a 1.2 µm pore size filter to avoid dust contaminants is recommended. The other issue is that peak particle size determined in this study, the modal diameter of the number distribution d*N*/d*D_p_*, is not the same as 〈*D_h_*〉*_Z_*, the particle size measured by dynamic light scattering. Based on the full size distribution for the 60 nm spheres and the 100 nm spheres, which are given in Table 21, the number median, number mean, the volume mean, the light scattering mean, as well as the DLS mean diameter were computed using a trapezoidal rule approximation. The mean results given in Table 22 indicate that for both the 60 nm and 100 nm spheres, the value of 〈*D_h_*〉*_Z_* is within 1 nm of the peak particle size. The values of 〈*D_h_*〉*_Z_* determined from the DMA measurements, 101.3 nm and 59.7 nm, are close to the values obtained by DLS, 99.8 nm and 60.5 nm. The value of 〈*D_h_*〉*_Z_* obtained by Duke Scientific using DLS for the 100 nm spheres is 101 nm. The difference between the DLS measured size and the DMA result for 〈*D_h_*〉*_Z_* is within the nominal 2 % uncertainty of the DLS measurement.

For the 100 nm spheres with a relatively narrow distribution, the difference between the number mean diameter and the static light scattering mean, which is weighted by *D*^6^, is about 0.8 nm, while the difference for the more broadly distributed 60 nm spheres is about 4.5 nm. The number mean diameter for the 100 nm spheres was found to be 100.6 nm (Table 22) compared to Duke Scientific results of 100.7 nm obtained by DMA and 98.9 nm by TEM.

A third application of the SRM particles is in calibrating DMA’s. Using the DMA, the peak value of an unknown particle size can be determined from the peak in the voltage distribution of the unknown particle and the peak of the calibration standard as discussed in Sec. 2.2.2. There is a slight difference in the peak of the size distribution and the diameter corresponding to the peak in the mobility or voltage distribution. We have estimated this difference starting with an assumed Gaussian distributions for both the 60 nm sphere and the 100 nm spheres with peak sizes of 100.76 nm and 60.79 nm. The assumed standard deviations were 2.5 nm and 4.9 nm, both of which are close to the measured standard deviation near the peak. The transfer function integral, Eq. (12), was used to compute the number concentration exiting the DMA as a function of voltage. The peak voltage was determined, the corresponding mobility was calculated using Eq. (15), and finally the particle diameter was obtained by solving Eq. (2) using the iterative method described in Appendix A. In Table 23 the value of the assumed or “true” diameter is compared with the “measured” diameter for the 60 nm and 100 nm spheres for a sheath flow of 20 L/min and for aerosol flows of 0.5 L/min, 1 L/min, and 2 L/min. The difference between “true” peak diameter and the “measured” diameter is about 0.07 % for the 100 nm spheres and about an 0.8 % overestimate for the “measured” diameter for the 60 nm spheres. As seen in Table 23, the flow ratio has little effect on the “measured” peak size using this approximate method. The difference is a factor of ten greater for the much broader 60 nm spheres, but the difference is still well within the requirement of SEMI Standard M52 [38], which requires a peak size measurement with an expanded uncertainty of at most 3 %. As shown in Sec. 2.2.3 and Table 1, a more accurate estimate of the peak size for broad distributions is obtained using Eq. (14) rather than using the peak in the concentration *vs* voltage.

A fourth application of calibration particles with a significant technological importance is in the production of deposition standards for surface scanning inspection systems used by the semiconductor industry. The fabrication of deposition standards is similar to the measurements discussed in the preceding paragraph. First the peak in the voltage distribution is located and then, particles from the monodisperse flow are electro-statically deposited onto a wafer. In this case one is also interested in the size distribution of the “monodisperse” aerosol exiting the DMA. The resulting size distribution is obtained from the following product where the transfer function is evaluated at the peak in the voltage distribution:

$$G_{mono}\left(D_{p}\right)=\Omega \left(Z_{p}\left(D_{p}\right),V_{peak}\right)G\left(D_{p}\right)p\left(D_{p}\right).$$ (58)

**Fig. 21:**
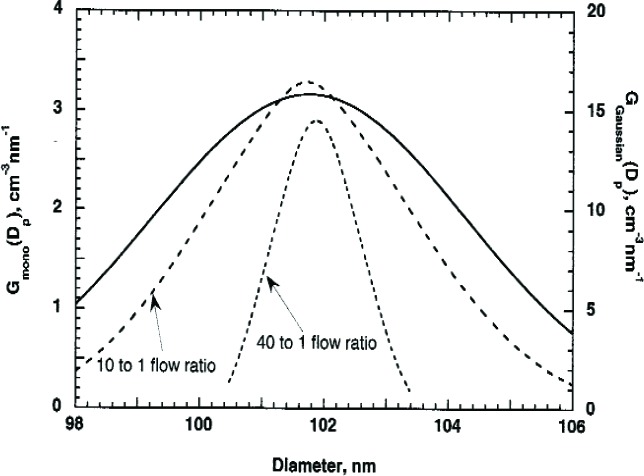
The calculated size distribution of the “monodisperse” aerosol exiting the DMA is shown for DMA flows of 20 L/min sheath and 2 L/min aerosol flow (dashed curve) and 0.5 L/min aerosol flow (dotted curve). The assumed Gaussian size distribution of the 100 nm spheres entering the DMA (peak diameter 100.76 nm and standard deviation of 2.5 nm) is shown by a solid line.

**Fig. 22:**
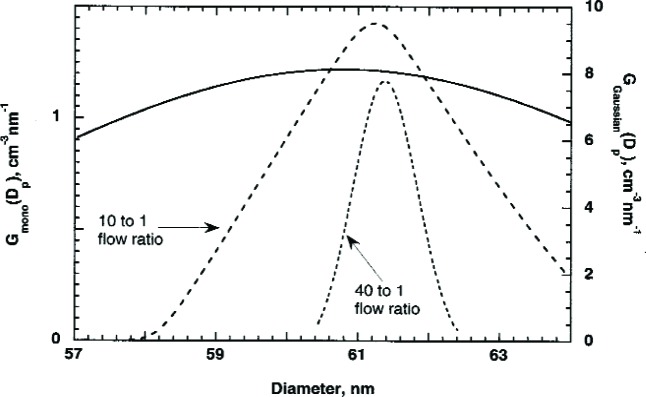
The calculated size distribution of the “monodisperse” aerosol exiting the DMA is shown for DMA flows of 20 L/min sheath and 2 L/min aerosol flow (dashed curve) and 0.5 L/min aerosol flow (dotted curve). The assumed Gaussian size distribution of the 60 nm spheres entering the DMA (peak diameter 60.79 nm and standard deviation of 4.9 nm) is shown by a solid line.

The size distribution function *G_mono_*(*D_p_*) is computed assuming the same input size distribution as the previous paragraph. The transfer function *Ω* includes the Brownian motion of the spheres [24,25]. The resulting size distributions shown in Figures 21 and 22 are similar in shape to the transfer functions except for the case of the 100 nm spheres and a 10 to 1 flow ratio where the width of the size distribution and the transfer function are similar. The effect of diffusion is to round the peak and broaden the distribution at the base. Without Brownian motion, the distributions would have a nearly triangular shape with a peak independent of the flow ratio. The Brownian motion is responsible for the decrease in the peak of the size distribution for the larger flow ratio. The exit size distribution for the 60 nm spheres for a flow ratio of 10 to 1 is slightly broader than the requirements of SEMI Standard M52 [38] which require a ratio of full-width-at-half-maximum (FWHM) to the peak particle size of 5 %. This ratio for the 60 nm spheres is predicted to be 5.5 %.

When using the 100 nm spheres or the 60 nm spheres for calibrating another particle sizing instrument, the uncertainty in the diameter of calibration spheres is part of the overall uncertainty in the particle sizing instrument. In this case the combined uncertainty, *u_combined_*, (Sec. 8.4) is used in estimating the uncertainty. The values for the 100 nm and 60 nm spheres are 0.55 nm and 0.31 nm. The number of degrees of freedom (Sec. 8.4) for the 100 nm uncertainty is 153 and for the 60 nm spheres is 123. These values may be needed in estimating the effective degrees of freedom for the particle sizing instrument.

### 11.2 Uncertainty Issues for Smaller Particle Sizes

The uncertainty in the calibration diameter is more than a factor of 2 larger than the next largest uncertainty term. Thus, an improved calibration diameter would reduce the overall measurement uncertainty of smaller particles based on DMA measurements. Ehara *et al*. [39] measured the number average diameter of SRM^®^ 1963 and obtained a value of 100.8 nm with an expanded uncertainty of 0.66 nm compared to the NIST certified uncertainty of 0.95 nm. Adopting the results by Ehara *et al*. might reduce the uncertainty in the 100 nm and 60 nm PSL spheres by 20 % to 25 %.

The measurement of angle resolved light scattering by particles on the surface [40] has resulted in an estimated relative combined Type B uncertainty of about 0.3 % for 100 nm spheres compared to the DMA value of about 0.5 %. However, there is about a 5 % difference between the particle size by light scattering versus the mobility measurement [41]. This difference is thought to be at least partly a result of deformation of the particle on the surface. Work is in progress to address this issue. Other promising approaches for obtaining low uncertainties include small angle x-ray scattering [42,43], field flow particle fractionation followed by angle resolved light scattering [44], and transmission electron microscopy [37].

Lastly we consider additional major contributors to the uncertainty in using the DMA sizing method for smaller particle sizes. The slip correction was not a dominant uncertainty for the 60 nm and 100 nm spheres because of the correlation between the uncertainty in the slip correction parameter *A* for the unknown and for the calibration particle. This effect will continue for particles as small as 20 nm, but for Knudsen numbers corresponding to particle sizes less than about 10 nm at 101.3 kPa, the slip correction was determined using a different calibration particle from the one used for the larger particles. In this case little correlation may be assumed between the two slip corrections.

For particle diameters smaller than 30 nm, Brownian motion has a significant effect on the transfer function. As indicated in Table 15, the width of the transfer function increases by a factor of 1.65 for a 30 nm sphere and by a factor of 3.99 for a 10 nm sphere compared to the case with no diffusion. There is also a large decrease in the peak of the transfer function from unity to 0.25 for the 10 nm spheres. For these cases a significant difference in the measured particle size is expected compared to the values with a triangular transfer function. The diffusion effects are also much greater for the flow ratio of 0.025 compared to 0.10 because of the much narrower flow volume trajectory to the outlet slit. One approach for reducing the diffusion effect is to use a Nano-DMA, which has about an 8 fold shorter length and a corresponding decrease in the time available for diffusion.

## 12. Summary

The peak size in the number distribution of the PSL aerosol and expanded uncertainty (95 % confidence level) for two new nanometer range size standards obtained using DMA measurements are 101.8 nm ± 1.1 nm and 60.39 nm ± 0.63 nm. The estimated standard deviations of the size distributions are approximately 2.5 nm for the 100 nm spheres and 4.9 nm for the 60 nm spheres. The average diameters based on number weighting, mass weighting, light scattering weighting, and dynamic light scattering weighting are presented.

The peak mobility measurements of samples selected at random from the 150 bottles indicate that the samples are homogeneous. The dynamic light scattering measurement showed no evidence of agglomeration and provided results consistent with the DMA measurements. In addition, the dynamic light scattering measurements will be used periodically in the future to verify that multimers are not forming in the samples.

Key features of the measurements to provide low uncertainty were an accurate size calibration standard; a modified sheath/aerosol inlet, a high ratio of the sheath flow to the aerosol flow (40 to 1), and a recirculating sheath flow; the use of the transfer function integral in the calibration process and in validating the accuracy of the inferred peak particle size; accurate pressure, temperature, and voltage instrumentation; the use of electrospray to avoid multimers and reduce the effect of nonvolatile residue for the 60 nm spheres; correcting for the linear drift in the concentration produced by the electrospray and accounting for the presence of doublet produced by the pneumatic nebulizer. An important consideration in the uncertainty analysis was the correlation between the slip correction of the calibration particle and the measured particle. This reduced the expanded uncertainty from about 1.8 % of the particle size to about 1.0 %.

The peak diameter is for the PSL spheres as an aerosol. The contaminants in the PSL suspension result in a residue layer on the spheres. The estimated thickness of this layer is 0.30 nm for the 100 nm spheres and 0.03 nm for the 60 nm spheres. The layer thickness for a fixed particle suspension was shown to be strongly dependent on the method of generating the aerosol. The change in layer thickness for the SRM^®^ 1963 spheres for the different generators resulted in a change in the peak PSL aerosol particle size for the 100 and 60 nm spheres and is also the second largest source of uncertainty for these measurements. The change in the peak particle diameter for the 100 nm spheres was + 0.11 nm with an uncertainty of ± 0.21 nm and for the 60 nm spheres was − 0.11 nm with an uncertainty of ± 0.19 nm.

The application of the 60 nm and 100 nm calibration particles in calibrating a DMA for sizing measurements and as deposition standards was discussed. It was shown that depositing the calibration particles with a DMA at the peak voltage using a flow ratio of 10 to 1 will meet the particle sizing accuracy requirement of SEMI Standard M52 though the size distribution is slightly broader for the 60 nm case than the required 5 % full-width-half-maximum. The other two flow ratios of 20 to 1 and 40 to 1 result in size distributions exceeding the requirements of the SEMI Document.

## Figures and Tables

**Fig. 23:**
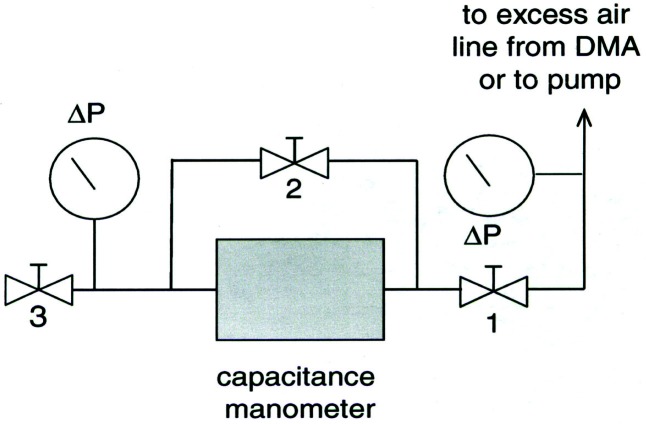
Experimental set up for the pressure measurements.

**Firg. 24:**
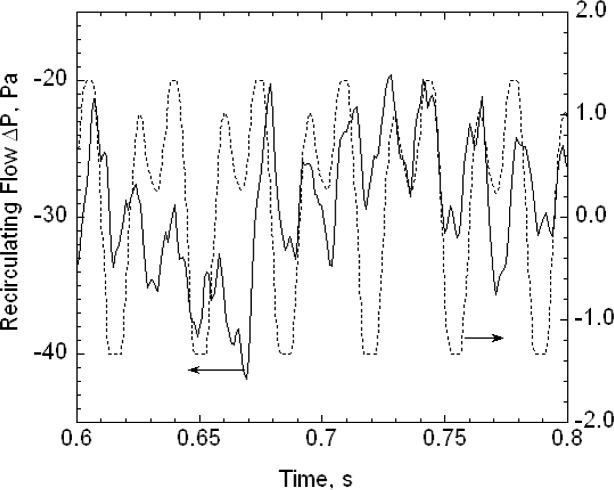
The pressure difference is plotted as a function of time for the recirculating flow (solid line) and for the pump flow (dashed line). The pump pressure exceeds the 1.33 kPa range of the pressure transducer.

**Fig. 25:**
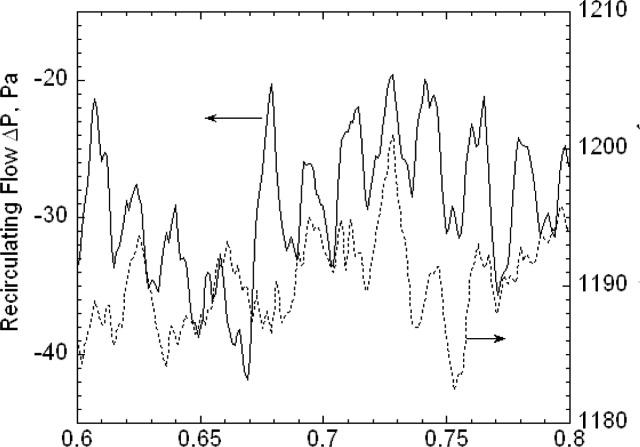
The pressure difference is plotted as a function of time for the recirculating flow (solid line) and for the steady flow from the N2 cylinder (dashed line).

**Fig 26-upper:**
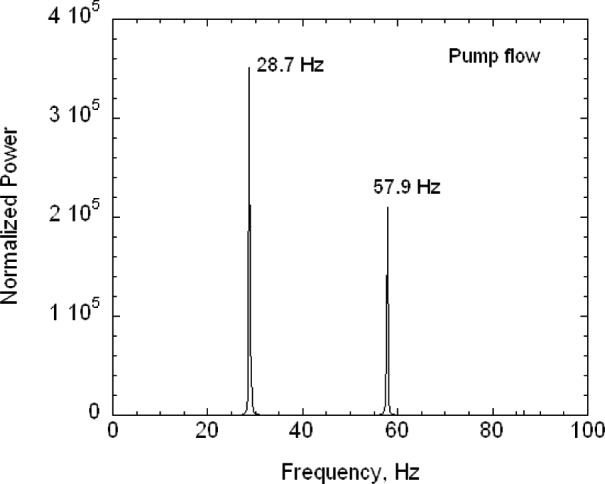
The power spectra obtained from the pressure data are plotted for the pump flow(upper), for the recirculating flow (middle), and for flow from a N_2_ cylinder (lower). In all three cases the same two characteristic frequencies of 29 Hz and 58 Hz appear.

**Fig 26-middle:**
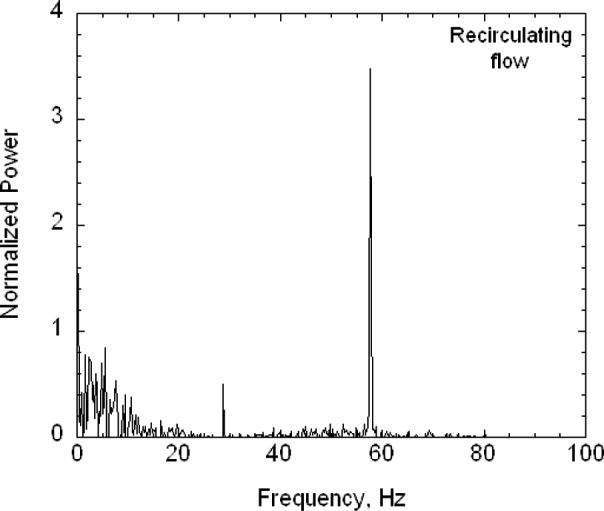
The power spectra obtained from the pressure data are plotted for the pump flow(upper), for the recirculating flow (middle), and for flow from a N_2_ cylinder (lower). In all three cases the same two characteristic frequencies of 29 Hz and 58 Hz appear.

**Fig 26-lower:**
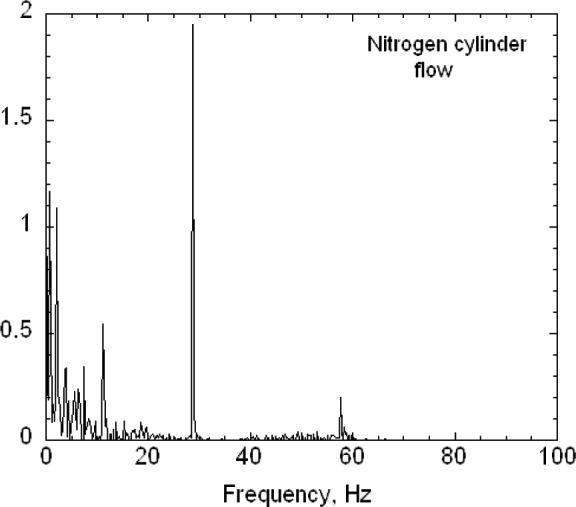
The power spectra obtained from the pressure data are plotted for the pump flow(upper), for the recirculating flow (middle), and for flow from a N_2_ cylinder (lower). In all three cases the same two characteristic frequencies of 29 Hz and 58 Hz appear.

**Table 1:** Comparison of diameters computed by approximate methods with the correct peak diameter

Assumed Peak diameter, nm	Peak diameter using method 1, nm	*D_p_*(1)−*D_p_*, nm	Peak diameter using method 2, nm	*D_p_*(2)−*D_p_*, nm
60.700	60.697	−0.003	61.133	0.433
101.600	101.577	−0.023	101.646	0.046

**Table 2:** Pressure drop inside classifier

Aerosol Flow Rate, L / min	Mean Pressure Drop, Pa	Standard Deviation of Mean, Pa
0.5	2.9	0.3
1.0	8.7	0.4
2.0	26.0	0.3

**Table 3:** Repeatability of 100 nm measurements

Date	Sample	File	Voltage Peak, V	*Vr*	Indices *i,j,k*
9/10/2004	50 Control	A	3501.5		
	92	B	3493.1	0.99760	1,1,1
	42	C	3493.1	0.99759	1,2,1
	92	D	3493.2	0.99676	1,1,2
	50 Control	E	3504.6		
	42	F	3506.4	1.00052	1,2,2
	102	G	3509.7	1.00130	1,3,1
	102	H	3510.2	1.00143	1,3,2
	50 Control	I	3505.2		

9/13/2004	50 Control	A	3537.5		
	93	B	3545.8	1.00235	2,1,1
	130	C	3554.1	1.00470	2,2,1
	25	D	3546.0	0.99995	2,3,1
	50 Control	E	3546.2		
	25	F	3551.5	1.00151	2,3,2
	130	G	3549.7	0.99993	2,2,2
	93	H	3543.0	0.99806	2,1,2
	50 Control	I	3549.9		

9/14/2004	50 Control	A	3552.8		
	4	B	3552.4	0.99989	3,1,1
	74	C	3549.9	0.99916	3,2,1
	34	D	3540.9	0.99860	3,3,1
	50 Control	E	3545.8		
	34	F	3548.5	1.00074	3,3,2
	4	G	3549.4	1.00161	3,1,2
	74	H	3547.1	1.00097	3,2,2
	50 Control	I	3543.7		

			Mean	1.00015	
	Relative standard uncertainty of mean, $u_{r}^{A}(\bar{V}r)$			0.0465 %	

**Table 4:** ANOVA table for nested, random two-factor analysis for 100 nm spheres

Sum of Squares	Degrees of Freedom	MS	E[MS]
SSTotal	(3 · 3 · 2)−1	SSTotal / [(3 · 3 · 2)−1]	Not Important
SSDay	(3−1)	SSDay / [(3−1)]	$\sigma_{\varepsilon }^{2}+3(2)\sigma_{\alpha }^{2}+2\sigma_{\beta }^{2}$
SSBottle(Day)	3 · (3−1)	SSBottle(Day) / [3 · (3−1)]	$\sigma_{\varepsilon }^{2}+2\sigma_{\beta }^{2}$
SSError	3 · 3 · (2−1)	SSError / [3 · 3 · (2−1)]	$\sigma_{\varepsilon }^{2}$

**Table 5:** Repeatability of 60 nm measurements

Date	Sample	File	Voltage Peak, V	*Vr*	Indices *i,j,k*
2/8/2005	38 Control	A	1387.4		
	39	B	1386.0	0.99899	1,1,1
	39	C	1390.5	1.00245	1,1,2
	38 Control	D	1387.1		
	38 Control	E	1386.6		
	120	F	1388.0	1.00101	1,2,1
	120	G	1392.4	1.00317	1,2,2
	38 Control	H	1388.0		

2/9/2005	38 Control	A	1391.4		
	1	B	1392.2	1.00057	2,1,1
	1	C	1385.1	0.99640	2,1,2
	38 Control	D	1390.1		
	38 Control	E	1400.6		
	134	F	1389.6	0.99215	2,2,1
	134	G	1388.0	0.99505	2,2,2
	38 Control	H	1394.9		

2/10/2005	38 Control	A	1394.7		
	117	B	1389.0	0.99591	3,1,1
	26	C	1406.1	1.01224	3,2,1
	38 Control	D	1389.1		
	38 Control	E	1384.7		
	117	F	1371.4	0.99040	3,1,2
	26	G	1376.5	0.99508	3,2,2
	38 Control	H	1383.3		

			Mean	0.99841	
	Relative standard uncertainty of mean, $u_{r}^{A}(\bar{V}r)$			0.2753 %	

**Table 6:** ANOVA table for nested, random two-factor analysis for 60 nm spheres

Sum of Squares	Degrees of Freedom	MS	E[MS]
SSTotal	(3 · 3 · 2)−1	SSTotal / [(3 · 2 · 2)−1]	Not Important
SSDay	(3−1)	SSDay / [(3−1)]	$\sigma_{\varepsilon }^{2}+3(2)\sigma_{\alpha }^{2}+2\sigma_{\beta }^{2}$
SSBottle(Day)	3 · (2−1)	SSBottle(Day) / [3 · (2−1)]	$\sigma_{\varepsilon }^{2}+2\sigma_{\beta }^{2}$
SSError	3 · 2 · (2−1)	SSError / [3 · 2 · (2−1)]	$\sigma_{\varepsilon }^{2}$

**Table 7:** Particle diameter, adjusted flow, *T*, and *P* for 100 nm spheres accurate sizing measurements

Date	Sample	Diameter, nm	Flow, L/min*	*T*(avg), °C	Pressure, kPa
9/16/2004	SRM1963		21.235	24.187	100.170
	B	101.54	**21.235**	24.234	100.162
	SRM1963		21.234	24.251	100.152
	D	101.48	**21.210**	24.269	100.136
	SRM1963		21.185	24.267	100.112
	F	101.57	**21.223**	24.295	100.101
	SRM1963		21.223	24.314	100.093

9/20/2004	SRM1963		21.102	24.025	101.130
	B	101.70	**21.102**	24.027	101.106
	SRM1963		21.153	24.060	101.092
	D	101.46	**21.126**	24.102	101.088
	SRM1963		21.099	24.139	101.077
	F	101.71	**21.149**	24.190	101.053
	SRM1963		21.149	24.215	101.032

9/22/2004	SRM1963		21.064	24.191	100.736
	B	101.59	**21.064**	24.205	100.732
	SRM1963		21.073	24.196	100.718
	D	101.64	**21.061**	24.191	100.697
	SRM1963		21.048	24.183	100.686
	F	101.67	**21.084**	24.155	100.669
	SRM1963		21.084	24.189	100.660

	Mean Diameter	101.60			
Relative standard deviation of mean, $u_{r}^{A}({\bar{D}}_{p})$		0.0300 %			

* The values in bold are based on the calibration data and are used to compute the peak diameter.

**Table 8:** ANOVA table for one factor analysis with random effects for 100 nm spheres

Sum of Squares	Degrees of Freedom	MS	E[MS]
SSTotal	(3 · 3)−1	SSTotal / [(3 · 3)−1]	Not Important
SSDay	(3−1)	SSDay / [(3−1)]	$\sigma_{\varepsilon }^{2}+3\sigma_{\alpha }^{2}$
SSError	3 · (3−1)	SSError / [3 · (3−1)]	$\sigma_{\varepsilon }^{2}$

**Table 9:** Particle diameter, adjusted flow, *T*, and *P* for 60 nm spheres accurate sizing measurements

Test Date	Test ID	Diameter, nm	Flow, L/min*	*T*(avg), °C	Pressure, kPa
2/15/2005	SRM1963		20.377	20.590	100.168
	B	60.451	**20.377**	20.713	100.140
	C	60.438	**20.398**	20.731	100.117
	SRM1963		20.398	20.744	100.100
	SRM1963		20.411	20.739	100.108
	F	60.435	**20.411**	20.707	100.084
	G	60.408	**20.417**	20.672	100.077
	SRM1963		20.417	20.649	100.066

2/16/2005	SRM1963		20.472	20.465	99.020
	B	60.525	**20.472**	20.491	99.073
	C	60.641	**20.427**	20.523	99.087
	SRM1963		20.427	20.563	99.164
	SRM1963		20.413	20.588	99.229
	F	60.615	**20.413**	20.638	99.257
	G	60.518	**20.457**	20.680	99.247
	SRM1963		20.457	20.746	99.396

2/18/2005	SRM1963		20.307	20.483	100.322
	B	60.155	**20.307**	20.556	100.332
	C	60.567	**20.306**	20.611	100.354
	SRM1963		20.306	20.651	100.396
	SRM1963		20.325	20.664	100.429
	F	60.277	**20.325**	20.649	100.488
	G	60.516	**20.322**	20.679	100.498
	SRM1963		20.322	20.618	100.498

	Mean Diameter	60.462			
Relative standard deviation of mean, $u_{r}^{A}({\bar{D}}_{p})$		0.0659 %			

* The values in bold are based on the calibration data and are used to compute the peak diameter.

**Table 10:** ANOVA table for one factor analysis with random effects for 60 nm spheres

Sum of Squares	Degrees of Freedom	MS	E[MS]
SSTotal	(4 · 3)−1	SSTotal / [(4 · 3)−1]	Not Important
SSDay	(3−1)	SSDay / [(3−1)]	$\sigma_{\varepsilon }^{2}+3\sigma_{\alpha }^{2}$
SSError	4 · (3−1)	SSError / [4 · (3−1)]	$\sigma_{\varepsilon }^{2}$

**Table 11:** Summary of Type A statistical analysis for particle sizing uncertainty

Experiment		Quantity	Average	Standard uncertainty	$u_{r}^{\text{Type}\;A}({\bar{D}}_{P})$	Degrees of Freedom
Repeatability,	100 nm	Dp (..)	101.60 nm	3.5E − 2 nm	0.066 %	17
Repeatability,	60 nm	$\bar{V}r\ldots$	0.99841	2.75 × 10^−3^	0.158 %	11
Certification,	100 nm	${\bar{D}}_{p}(..)$	101.60 nm	3.05E − 2 nm	0.030 %	8
Certification,	60 nm	${\bar{D}}_{p}(..)$	60.46 nm	3.98E − 2 nm	0.066 %	11

**Table 12:** Summary of Type B uncertainties contributing to the uncertainty in the 100 nm and 60 nm spheres

Quantity/Effect	Value	% uncertainty
*e*, electronic charge	1.6022 E − 19 kg m^2^ s^−1^ V^−1^	Negligible
*T*, temperature	296.15 K	0.03
*P*, pressure	101.33 kPa	0.01
*µ*, viscosity	1.8325 E − 5 kg m^−1^ s^−1^	0.09
$D_{p}^{s}$, SRM® 1963 diameter	100.7 nm	0.47
*V*, voltage		
SRM®1963 and 100 nm spheres	3400 V	0.10
60 nm spheres	1400 V	0.20
*G_f_*, geometric constant	0.60299	fixed value
*λ*_0_	67.30 nm	fixed value
Asymmetry in SRM size dist.		
100 nm spheres^a^		0.067
60 nm spheres^a^		0.054
Drift in electrospray, 60 nm^a^		0.060
Doubly charged trimers, 100^a^		0.079
Residue Layer		
100 nm spheres^a^		0.210
60 nm spheres^a^		0.150

a The uncertainties for these terms refer to the resulting uncertainty in the particle diameter.

**Table 13:** Slip correction parameter *A*^a^ uncertainty for *T* = 296.15 K and *P* = 101.33 kPa

*D_p_*, nm	*C*	$u_{r}^{A}(A)$	$u_{r}^{B}(A)$	*u_r_*	*u_r_* (*D_p_*; *A*, *A_s_*) With correlation	*u_r_* (*D_p_*; *A*, *A_s_*) Without correlation
100.70	2.8634	0.217 %	1.388 %	1.404 %		
101.60	2.8449	0.218 %	1.391 %	1.409 %	0.121 %	0.784 %
60.46	4.2807	0.199 %	1.239 %	1.251 %	0.107 %	0.750 %

a The quantities $u_{r}^{A}(A),\;u_{r}^{B}(A)$, and *u_r_* are the Type A uncertainty, the Type B uncertainty, and the combined uncertainty for the slip correction parameter *A*. The quantity *u_r_* (*D_p_*; *A*, *A_s_*) is the uncertainty in the particle diameter arising from the uncertainties in the slip correction parameter for the measured particle, *A*, and for the 100.7 nm SRM® 1963 particles, *A_s_*.

**Table 14:** Combined relative Type B uncertainty and components for Eq. (38)

*D_p_*, nm	Term 1, *D_p_* for SRM	Term 2, *V*	Term 3, *V* for SRM	Term 4, *A*, *A*_s_	Term 5, *T*	Term 6, *P*	*u_r_ (D_p_)*
101.60	0.471 %	0.061 %	0.060 %	0.121 %	5.3 E − 5 %	1.4 E − 5 %	0.494 %
60.46	0.439 %	0.113 %	0.057 %	0.107 %	0.009 %	6.5 E − 4 %	0.469 %

**Table 15:** Effect of particle diffusion on the peak height and dimensionless width of the transfer function

*D_p_*, nm	*Diff*, m2/s	Ω*_Diff_* (*peak*)*/*Ω*_NoDiff_* (*peak*)^a^	*σ_Diff_/*σ*_NoDiff_*^b^
10	5.52 E-08	0.25	3.99
30	6.41 E-09	0.59	1.65
60.7	1.68 E-09	0.78	1.21
101.6	6.62 E-10	0.78	1.09

a Correction factor to peak value of the transfer fraction, which occurs at *x* = 1, resulting from particle diffusion.

b Correction factor for the width of the transfer function resulting from diffusion.

**Table 16:** Bias correction for peak diameter of the 100 nm and 60 nm sphere sizes

*D_p_*, nm	*β*(asym), nm	*β*(residue), nm	*D_p_*(corr), nm
101.60	0.05	0.11	101.76
60.46	0.04	−0.11	60.39

**Table 17:** Total relative Type B uncertainty and components

*D_p_*, nm	$u_{r}({\bar{D}}_{p})$	*u_r_* (asym)	*u_r_* (drift)	*u_r_* (doublet)	*u_r_* (residue)	*u_r_* Total Type*B*
101.76	0.494 %	0.044 %		0.079 %	0.206 %	0.543 %
60.39	0.469 %	0.055 %	0.060 %		0.189 %	0.512 %

**Table 18:** Computation of expanded uncertainty

*D_p_*, nm	*u_r_*^Type^*^A^*	*ν*^a^	*u_r_*^TotalType^*^B^*	*u_combined_*	*ν_eff_*^b^	*k_p_*	*U_r_*(*D_p_*)
101.76	0.030 %	8	0.543 %	0.544 %	153	2.0165	1.10 %
60.39	0.066 %	11	0.512 %	0.516 %	123	2.0205	1.04 %

a Degrees of freedom for Type A uncertainty.

b Estimated degrees of freedom for *u_combined_*

**Table 19:** Dynamic light scattering results for 60 nm spheres

Sample Size/Number	Rate, s^−1^	⟨*D_h_*⟩*_Z_*, nm	Polidispersity fraction
**April 5, 2005**			
SRM1963, A1a	147 300	105.9 ± 1.0	0.037 ± 0.029
60 nm, 26b	71 500	63.4 ± 1.1	0.087 ± 0.048
60 nm, 134a	88 100	60.8 ± 1.4	0.062 ± 0.050
60 nm, 117a	95 400	59.5 ± 0.7	0.072 ± 0.024
SRM1963, A1b	115 100	104.1 ± 1.1	0.038 ± 0.032

**April, 6,2005**			
SRM1963, A1c	54 200	105.8 ± 1.2	0.116 ± 0.057
60 nm, 39a	92 600	59.3 ± 0.9	0.054 ± 0.040
60 nm, 1a	93 200	59.7 ± 0.9	0.053 ± 0.035
60 nm, 120a	95 600	60.4 ± 1.6	0.094 ± 0.046
SRM1963, A1d	101 600	104.0 ± 1.0	0.047 ± 0.019

Averages, 60 nm spheres		60.5 ± 1.5	0.070 ± 0.018

**Table 20:** Dynamic light scattering results for 100 nm spheres

Sample Size/Number	Rate, s^−1^	⟨*D_h_*⟩*_Z_*, nm	Polidispersity fraction
**October 20, 2004**			
SRM1963, A1a	405 000	102.9 ± 0.08	0.019 ± 0.011
100 nm, 25a (2 drops)	622 000	99.0 ± 0.8	0.017 ± 0.012
100 nm, 25b diluted	108 400	100.1 ± 1.3	0.055 ± 0.022
100 nm, 42a (1 drop)	165 500	99.9 ± 1.0	0.032 ± 0.022
100 nm, 130a (1 drop)	42 600	98.0 ± 1.7	0.100 ± 0.56
100 nm, 130b (2 drops)	559 000	99.4 ± 1.6	0.012 ± 0.007

Averages, 100 nm spheres^a^		99.8 ± 0.4	0.033 ± 0.022

a The calculation of the averages are based on 25b, 42a, and 130b. Sample 103a was not used in the average because of its large polydispersity relative to the other samples.

**Table 21:** Particle size distribution for the 60 nm and 100 nm spherical particles

60 nm particle distribution		100 nm particle distribution	
*D_p_*, nm	*G*(*D_p_*), cm^−3^nm^−1^	*D_p_*, nm	*G*(*D_p_*), cm^−3^nm^−1^
10.81	2.44	78.15	1.25
15.43	2.25	82.85	1.96
19.08	3.49	87.38	4.85
22.14	2.76	91.76	5.04
24.85	8.19	94.81	21.73
27.32	20.29	95.23	23.86
29.61	32.81	95.65	31.86
31.74	61.88	96.07	34.37
33.76	111.74	96.49	42.48
35.68	108.55	96.90	48.58
37.52	156.44	97.32	60.43
39.28	211.27	97.74	71.22
41.23	265.07	98.15	85.86
40.98	266.30	98.56	97.29
42.62	341.30	98.97	117.15
44.21	376.48	99.39	134.30
45.76	482.36	99.80	151.50
47.26	492.41	100.20	168.21
48.73	546.23	100.61	181.11
50.16	650.91	101.02	193.62
51.56	755.21	101.43	190.09
52.93	928.43	101.83	192.60
54.27	1071.90	102.23	184.39
55.59	1290.67	102.64	172.33
56.89	1475.79	103.04	158.96
58.16	1662.98	103.44	136.69
59.41	1804.61	103.84	116.93
60.65	1863.79	104.24	93.65
61.86	1758.20	104.63	76.19
63.06	1555.91	105.03	58.30
64.24	1146.43	105.43	42.87
65.40	724.39	105.82	31.19
66.55	395.01	106.22	22.61
67.69	170.46	106.61	16.15
68.81	58.63	108.12	6.78
68.81	58.63	111.98	1.23
69.92	24.25	115.77	1.40
71.01	11.54		
72.10	9.71		
73.17	4.25		
74.24	2.76		
75.29	3.43		
77.37	3.34		
79.40	3.57		
80.41	3.69		
81.41	2.99		
81.41	2.96		
83.38	1.99		
83.38	3.29		
83.38	2.30		
85.32	1.25		
87.24	0.43		
89.13	0.21		
90.99	0.10		
9283	0.07		

**Table 22:** Mean particle size based on size distribution for 100 nm and 60 nm spheres with the standard deviation of the size distribution.

Peak Diameter, nm	⟨*D_N_*⟩, nm	⟨*D_V_*⟩, nm	⟨*D_LS_*⟩, nm	⟨*D_h_*⟩*_Z_*, nm	*σ*_⟨_*_DN_*_⟩_
60.55	55.70	58.49	60.23	59.72	7.90
101.52	100.61	101.05	101.43	101.31	3.97

Number averaged diameter $\langle D_{N}\rangle =\frac{\int DG(D)dD}{\int G(D)dD}$					
Standard deviation of size distribution $\sigma ={\left(\frac{\int {\left(D-\langle D_{N}\rangle \right)}^{2}G(D)dD}{\int G(D)dD}\right)}^{1/2}$					
Volume or mass averaged diameter of $\langle D_{V}\rangle =\frac{\int DD^{3}G(D)dD}{\int D^{3}G(D)dD}$					
Light scattering weighted diameter (Raleigh limit) $\langle D_{LS}\rangle =\frac{\int DD^{6}G(D)dD}{\int D^{6}G(D)dD}$					
Dynamic light scattering diameter $\frac{1}{{\langle D_{h}\rangle }_{Z}}=\frac{\int \frac{1}{D_{h}}D_{h}^{6}G(D_{h})dD_{h}}{\int D_{h}^{6}G(D_{h})dD_{h}}$					

**Table 23:** Comparison of DMA Diameter based on peak voltage with peak in size distribution with sheath air flow of 20 L/min

*D_peak_*, nm for assumed *G*(*D_p_*)	*D_peak_*, nm Q_a_ = 0.5 L/min	*D_peak_*, nm Q_a_ = 1.0 L/min	*D_peak_*, nm Q_a_ = 2.0 L/min
101.76	101.83	101.79	101.69
60.79	61.36	61.31	61.23

## Footnotes

1 Certain commercial equipment, instruments, or materials are identified in this paper to specify adequately the experimental procedure. Such identification does not imply recommendation or endorsement by the National Institute of Standards and Technology, nor does it imply that the materials or equipment identified are necessarily the best available for the purpose.

## Appendix A. Iterative Solution of Eq. (2) for Particle Diameter

In Sec. 2, the following expression is given for the electrical mobility as a function of particle diameter:

$$Z_{p}=\frac{eC(Kn)}{3\pi \mu D_{p}}.$$ (A1)

Given the nonlinear dependence of the Cunningham slip correction parameter on the Knudsen number, and hence the particle diameter, a closed form expression for the diameter can not be obtained from Eq. (A1).

An iterative technique is, therefore, used to solve for the particle diameter. Given an initial estimate for the particle diameter, *D*_old_, the slip correction parameter is calculated. A new estimate for the particle diameter is then calculated by rearranging Eq. (A1),

$$D_{\text{new}}=\frac{eC(Kn(D_{\text{old}}))}{3\pi \mu Z_{p}}.$$ (A2)

A convergence check is performed and, if the two diameters are determined to not have converged, the value of *D*_old_ is updated according to the expression,

$$\frac{1}{2}(D_{\text{new}}+D_{\text{old}})\to D_{\text{old}}.$$ (A3)

The process is then repeated until convergence is obtained.

Given a good initial estimate, convergence to within O(10^−7^) using the above technique is typically achieved within approximately 8 iterations. The initial estimate is obtained by neglecting the exponential term within the slip correction factor.

$$C=1+2\lambda A_{1}/D_{p},$$ (A4)

which, when substituted into Eq. (A1), allows a closed form initial estimate for the diameter,

$$D_{\text{init}}=\frac{e}{6\pi \mu Z_{p}}\left(1+\sqrt{1+\frac{6\pi A_{1}\lambda \mu Z_{p}}{e}}\right).$$ (A5)

## Appendix B. Measurement of Pressure Fluctuations in the DMA Flow

In using the DMA to measure the size distribution of aerosols it is important that the flow be laminar. The recirculation flow used with the DMA in this study includes buffer tanks to remove the pressure fluctuations and the corresponding flow fluctuations. To assess the effectiveness of the buffer tanks, the pressure in the excess flow line just outside the DMA chassis (Fig. 3) was measured with a capacitance type differential pressure gauge. The specific instrument used was a 10 torr MKS Baratron Model 698A11TRA pressure transducer operated at a 1 ms time constant setting.

The key features of the measurement illustrated in Fig. 23 include a valve to null the pressure difference across the MKS gauge, valves to isolate the two sides of the MKS gauge, and Gardon type pressure gauges on each side of the MKS gauge for monitoring the value of the pressure relative to the ambient pressure. Valve 2 is normally in the open position to short the two sides of the pressure transducer to avoid an overpressure damaging the gauge. After allowing a couple hours for the electronics to warm up, valve 3 is closed and valve 1 is opened. At this point the pressure difference is close to zero with a value of about ± 0.1 Pa. Next, valve 2 is closed so that one side of the pressure transducer has a constant pressure from the gas confined by the valves while the pressure on the other side has nearly the same average pressure, but it also has any pressure fluctuations that might be present from the pump. This configuration with a compensating pressure on one side allows the use of a sensitive gauge with a maximum range of 1.33 kPa (10 torr) even though the pressure difference from ambient is as large as 2.7 kPa. The pressure data is typically collected at a speed of 1 kHz for 10 s.

Four sets of measurements were carried out. In the first set the pressure gauge was connected to the system in its normal configuration with a recirculating flow of 20 L/min. In this case the average pressure at the inlet side of the pump is − 1.7 kPa relative to the ambient pressure. In the second set, a nitrogen gas cylinder is used to provide a steady flow of 20 L/min through the DMA. In this case the cylinder flow is connected to the sheath flow inlet and the excess flow to an exhaust line. The recirculating system is disconnected from the flow. This measurement was performed to observe the pressure fluctuations for a steady flow. In this case, the average pressure is about 0.61 kPa above ambient. The fluctuations from the pump are observed in the third set of experiments by connecting the outlet of the two pumps (Thomas 107 CAB18 diaphragm type) to the pressure transducer. In this case the average pressure is 2.7 kPa above ambient. In all three cases, the 0.79 cm (5/16 in) ID tubing was unobstructed for at least 40 diameter before and after the tee for the pressure measurement. The 60 cm of tubing and connectors/valves between the gauge and the pressure transducer were identical for the steady flow and the recirculating flow. For the pump measurements, the length of tubing was increased by about 25 cm. The fourth measurement was with no flow with valve’s 1 and 3 closed and 2 open. It was designed to measure the electronic nose of the gauge.

The electronic noise was small with a standard deviation based on 1 s of data of about 0.25 Pa. The pressure fluctuation from the pump has a distinct frequency of about 29 Hz and with an amplitude in excess of ± 1333 Pa (10 torr) as shown in Fig. 24. There is a secondary peak occurring at the same frequency. The pressure fluctuation for the recirculating flow appear to correlate somewhat with the pressure peaks including the secondary peaks for the pump alone as indicated in Fig. 24. The nominal magnitude of the fluctuations is about 5 Pa about the mean which is at least 270 times smaller than the amplitude of the pump pressure.

The pressure fluctuation for the steady flow from the cylinder appear less regular than for the recirculating flow as shown in Fig. 25. The standard deviation of the pressure for a 1 s time period for the steady flow is about 3.7 Pa compared to a value of about 5.1 Pa for the recirculating flow. The fluctuations in the buffered flow is greatly reduced relative to the pump flow though the fluctuation pattern is more regular than for the steady flow and the noise level as indicated by the standard deviation is about 40 % larger than the steady flow.

To provide more quantitative information of the presence of characteristic frequencies, the power spectra for the pressure data were computed. Before computing the power spectrum, the average value of the pressure was subtracted from the pressure data so that the analysis was carried out for *P_v_*, the variation about the mean. The fast Fourier transform, *C*(*k*), was computed from the time sequence, and from this the norm of the amplitude was determined as a function of frequency.

$$\begin{array}{l} \hat{\Pi }(0)={\left({\left|C(0)\right|}^{2}/N\right)}^{2}\\ \hat{\Pi }(k)=2{\left({\left|C(k)\right|}^{2}/N\right)}^{2}\;\text{for}\;k=1,2,\ldots ,(N/2-1)\\ \hat{\Pi }(k=N/2)={\left({\left|C(k)\right|}^{2}/N\right)}^{2}. \end{array}$$ (B1)

The quantity *k* is a dimensionless frequency, which is related to frequency *f* by the following expression:

f=k/(4092Δt). (B2)

There were 4092 points data points analyzed and the time increment *Δt* was 0.001 s. The normalization in Eq. (B1) is such that the sum of the values of $\hat{\Pi }(k)$ is a measure of the variance of the pressure signal.

The power spectra plotted in Fig. 26 all have peaks near 30 Hz (28.7) and near 60 Hz (57.9). The power is about 1.0 × 10^5^ larger amplitude than for the recirculation flow. This again shows that the amplitude is greatly diminished for the recirculation flow. This result is consistent with the pressure measurements, since the power is proportional to the square of the pressure amplitude. The peaks near 30 Hz and 60 Hz for the nitrogen tank flow are surprising since the pump is not in the system. We do not have an explanation for this observation.
